# Capturing multiple interaction effects in L1 and L2 object-naming reaction times in healthy bilinguals: a mixed-effects multiple regression analysis

**DOI:** 10.1186/s12868-020-0549-x

**Published:** 2020-01-17

**Authors:** Severin Schramm, Noriko Tanigawa, Lorena Tussis, Bernhard Meyer, Nico Sollmann, Sandro M. Krieg

**Affiliations:** 1 Department of Neurosurgery, Klinikum rechts der Isar, Technische Universität München, Ismaninger Str. 22, 81675 Munich, Germany; 2 Faculty of Linguistics, Philology & Phonetics, University of Oxford, Walton Street, Oxford, OX1 2HG UK; 3 Department of Diagnostic and Interventional Neuroradiology, Klinikum rechts der Isar, Technische Universität München, Ismaninger Str. 22, 81675 Munich, Germany; 4 TUM-Neuroimaging Center, Klinikum rechts der Isar, Technische Universität München, Munich, Germany

**Keywords:** Bilinguals, Language, Object naming, Voice latency, Voice onset measurements, Word production

## Abstract

**Background:**

It is difficult to set up a balanced higher-order full-factorial experiment that can capture multiple intricate interactions between cognitive and psycholinguistic factors underlying bilingual speech production. To capture interactions more fully in one study, we analyzed object-naming reaction times (RTs) by using mixed-effects multiple regression.

**Methods:**

Ten healthy bilinguals (median age: 23 years, seven females) were asked to name 131 colored pictures of common objects in each of their languages. RTs were analyzed based on language status, proficiency, word choice, word frequency, word duration, initial phoneme, time series, and participant’s gender.

**Results:**

Among five significant interactions, new findings include a facilitating effect of a cross-language shared initial phoneme (mean RT for shared phoneme: 974 ms vs. mean RT for different phoneme: 1020 ms), which profited males less (mean profit: 10 ms) than females (mean profit: 47 ms).

**Conclusions:**

Our data support language-independent phonological activation and a gender difference in inhibitory cognitive language control. Single word production process in healthy adult bilinguals is affected by interactions among cognitive, phonological, and semantic factors.

## Background

Bilinguals and the language phenomena specific to them have long puzzled researchers, due to their deviation from monolinguals in multiple regards. For the process of word production in monolinguals, there is a general understanding of a sequential process that a person performs when naming an object. After first defining the concept to be expressed, a lemma is selected, a phonological code is retrieved, syllabified, and phonetically encoded before articulation ensues. This model was derived from a body of research that has identified specific time windows for each single step in word production [1, 2]. However, there are competing hypotheses to the proposed serial models. Indeed, some studies argued for cascade models in which a set of semantic candidates unselected could enter into the phonological stage and the corresponding multiple phonological codes are activated [3, 4].

Bilinguals show behavior yet to be fully explained by the current models. Compared to monolinguals, they possess a slower reaction time (RT) when confronted with an object-naming task, both in their first language (L1) and their second language (L2). Also, responses given in the L1 generally happen faster than in the L2 when L1 is of currently dominant use, but the reverse pattern has also been observed [5–7]. By now, a multitude of competing explanation attempts focusing on different specific steps of the word production process exist [5, 8].

Regarding the selection of the task-relevant language, phonological activations were shown to occur both in L1 and L2, suggesting that task-relevant language selection does not occur in the semantic/lemma selection stages [9, 10]. The inhibitory control (IC) model introduced the selection of task-relevant language earlier at the stage of semantic/lemma selection [11]. Herein, lexical representations are equipped with a mark indicating the corresponding language. A higher-level control system would then, depending on the task, inhibit all representations with the L1 or L2 mark respectively (resulting in effectively a L1- vs. a L2-mode), allowing for the correct lexical route to be taken [11]. Because these language modes would hardly ever be used equally, different levels of basal activation would result and make activation of one of the two languages more time-consuming.

Concerning variables related to the semantic/lemma selection stage, both language proficiency and age of acquisition of L2 have been shown to impact the RT. At present, it has been hypothesized that both earlier acquisition and higher proficiency can lead to stronger activation levels of lemmas and thereby faster RT, and that this effect may arise out of a modulation of cortical activity patterns, making the L2 activity progressively more (or less) similar to the L1 activity [5, 12, 13]. Because these findings stress the influence of lemma activation level on RT, we formed the hypothesis that obvious responses to a given stimulus (the modal response) should be given faster than less obvious responses (the non-modal response), due to the latter case reflecting a conflict in lemma selection, which would add time to the RT.

At the stage of phonological code retrieval, the word frequency (WF) effect must be mentioned. This phenomenon describes the tendency that the RT length diminishes when the target word is a commonly used one [14]. Currently, research indicates that the WF effect occurs relatively late in the word production process and at least partly reflects the process of phonological code retrieval [15]. A hypothesis on bilingual word-production delay focused on the WF effect is represented by the weaker-links hypothesis, which will be examined more closely later.

For an effect again more exclusive to bilinguals, we have to consider the language-independent, simultaneous activation of L1–L2 phonological representations that multiple studies point to [9, 10]. Such an activation implies a conflict having to be resolved between the L1 and L2 in choosing the phonological code in the task language. This would then impact the RT. In contrast, a shared initial phoneme increases the activation of the target phonological code, yielding a facilitating effect on RT. As a result, another hypothesis was formed for our present paper: analogous to one of the experiments performed by Colomé and Miozzo, we would expect a facilitating effect on RT when comparing target words that share the same initial phoneme between both their L1–L2 translations with target words that do not, arising out of an additive activation of the shared phoneme [10].

After the phonological code is retrieved and syllabification and phonetic encoding are applied, articulation ensues. Bilinguals herein commonly deal with differences in articulation efforts, because many times the different translations of a given target word contain varying numbers of syllables or even just differences in pronunciation, which impact the plan for forming the corresponding sound sequences. For example, German target words include more complex affricate “pf” or “ts” sounds that take longer than a simple obstruent “p”, which is rather prevalent in other languages. These and similar duration differences among phonemes in speech-motor planning influence the effort of articulation and cannot be captured by the number of phonemes, but could be assessed by measuring the actual word duration (WD).

Furthermore, we may not forget the importance of higher-level executive functions. New meta analyses seem to indicate that the widely presumed bilingual advantage over monolinguals for executive functions may in fact be less powerful than previously assumed, or even an artifact due to publication bias [16]. Considering this, findings suggesting such a possible positive effect of bilingualism on executive function should be reconsidered [17]. This justifies investigating the possibility of a bilingual disadvantage in some regards. As has been previously put forth, bilingualism may be connected to the expenditure of additional cognitive resources due to a higher need for self-monitoring during speech compared to monolinguals [18]. This might enable a fatigue effect, slowing down RT over time. Our study’s specific setup allowed for not only an analysis of such a fatigue effect, but also a learning effect and a possible interaction of both. Moreover, we formulated the hypothesis that due to motivational factors, an inverse relationship between accuracy in an object-naming task and RT is possible. This is based on the scenario of a speed-accuracy tradeoff that participants face when making the decision of either putting sustained effort into finding the correct response or, instead, focusing on minimizing the respective RT.

Another way in which the effects of bilingualism on higher cognitive functions have been evaluated is with the Simon task. Recently, a gender-dependent executive effect has been pointed out, with females being more easily distracted by the unnecessary spatial information presented during the task [19]. Translating this finding to a within-bilingual framework, it remains to be seen whether a similar gender effect can be shown in the context of bilingual language control regarding the suppression of task-irrelevant language. The respective hypothesis we defined in the current study was based on the previously mentioned effect of a cross-linguistically shared initial phoneme. If a gender-dependent cognitive-control advantage of suppressing the phonological information in the task-irrelevant language exists for males, they should profit less from the facilitating effect of a cross-linguistically shared initial phoneme compared to females.

Other important variables modulating cognitive control in different tasks are represented by age and age of L2 acquisition [5, 20]. Bilingualism has been linked to improved retention of cognitive skills in later life periods compared to monolingualism [21]. Furthermore, inhibitory cognitive control decreases with age as shown by the existing literature [21, 22]. This decrease would take effect in tasks relying on inhibitory control, such as finding appropriate non-modal responses when no modal response is present. Thus, one would therefore expect an increase in the difference in RT between modal- and non-modal responses with age. Regarding age of L2 acquisition, studies have struggled to clearly identify both the locus of influence as well as the mechanism of mediation [5]. Its connection to cognitive control mechanisms has hardly been explored, even though it is argued that there is a fundamental difference in network organization based on this variable [23]. Furthermore, age of L2 acquisition has been theorized to determine the size of phonological representations, with earlier learned words saved as blocks and expressions acquired later being deconstructed into phonological elements [24]. Thus, we hypothesize that the influence of the initial phoneme status (shared or different between L1 and L2) on RT would be stronger the later the L2 was acquired.

Importantly, there is not only the possibility of these factors acting isolated, but rather in combination with one another. Here one has to point toward the weaker-links hypothesis, according to which bilinguals possess a weaker connection between their semantic and phonological representations when compared to monolinguals. This is believed to occur due to the former having to split their phonological activations between two different sets of representations due to language specificity of phonological codes, while the latter are able to focus the entire activation on one single set [8, 25]. The hypothesis in this regard bears similarities to the theoretical underpinnings of the WF effect, but with the degree of the WF effect varying with language use. WF initially benefits high-frequency words, but eventually the low-frequency words catch up. Thus, it is hypothesized that RT difference between the high-frequency words and the low-frequency words would be smaller for the language of longer use than for the language of shorter use, and RTs would be shorter for the language of the currently dominant use than for the language of the currently non-dominant use. With regards to this theoretical construct, we set out to investigate the interaction between language dominance and WF on one hand and participant age and WF on the other hand.

In addition to the predictions we derived so far from the serial model extended to bilinguals, testing an interaction effect between word choice (WC; semantic/lemma selection) and phonological encoding in a single language (German) could distinguish the purely serial model, in which phonological encoding occurs only after semantic/lemma selection, from cascade models, in which a set of unselected semantic/lemmata activates phonological codes of these candidate lemmata [4]. Cascade models typically predict that WF effects increase when multiple lemma candidates remain unselected, which may be the case when non-modal word response is made, compared to when a modal word response is made [3]. To investigate the influence of the mentioned variables, the present study uses mixed-effects multiple regression and intends to test the effects of various established psycholinguistic and cognitive factors and new two-way interactions between these established factors in one statistical approach [26].

## Methods

### Participants and study design

The entire data used was collected from twenty healthy volunteers (median age: 24 years, 10 females; Table 1), 10 of which were collected in the context of a study on cortical language representations investigated by navigated transcranial magnetic stimulation (nTMS) [27]. The additional 10 volunteers were collected for analyses 3 and 4 (see below). The participants confirmed to the Kohnert definition of bilingualism, as each of them reported regular exposure to both L1 and L2 before the age of 10 years [28]. The following inclusion criteria were considered: age of at least 18 years, right-handedness according to the Edinburgh Handedness Inventory, and acquisition of two languages before the age of 10 years [27]. The data collection took place on two appointments separated by at least 14 days to exclude nTMS aftereffects [27]. In the present study, we utilized the data taken as “baseline”, meaning that object-naming performance prior to nTMS application was analyzed.

**Table 1:** Cohort characteristics

Volunteer	L1	L2	Age	Age of L2 acquisition
1	Italian	German	23	0
2	German	Italian	27	3
3	Slovakian	German	19	5
4	Chinese	German	25	5
5	Slovakian	German	25	10
6	Chinese	German	23	6
7	English	German	24	2
8	French	Luxemburgish	22	3
9	Luxemburgish	Cantonese	23	0
10	Luxemburgish	German	23	0
11	Luxemburgish	German	23	6
12	Luxemburgish	German	24	5
13	Luxemburgish	German	24	5
14	German	Italian	22	1
15	German	Spanish	30	1
16	Croatian	German	32	5
17	Luxemburgish	German	27	6
18	Bosnian	German	29	3
19	Croatian	German	31	6
20	Spanish	German	32	2

This table shows details on the first language (L1) and second language (L2) of the included participants. Age of the participants and age of L2 acquisition are given in years

### Object-naming task

The same object-naming task was carried out on both appointments (one per language, two consecutive runs per appointment) using a NexSpeech module (version 4.3; Nexstim Plc., Helsinki, Finland) [27]. It consisted of 131 colored pictures of different concrete animate and inanimate objects (such as “baby”, “rake”, or “orange”) in a sequence randomized for each run [27, 29–31].

During each appointment, the language used in the task was invariant. The sequence of languages was counterbalanced. Each participant was instructed to name the pictured object as simply, quickly, and plausibly as possible [27, 29–31]. One initial object-naming run was followed by another containing only the objects that the investigator deemed correctly named in the first run. The objects were displayed for 700 ms each, with an interval of 2500 ms between the display of two consecutive objects [27, 29–31].

### Audio extraction and measurement of reaction times

**Fig. 1:**
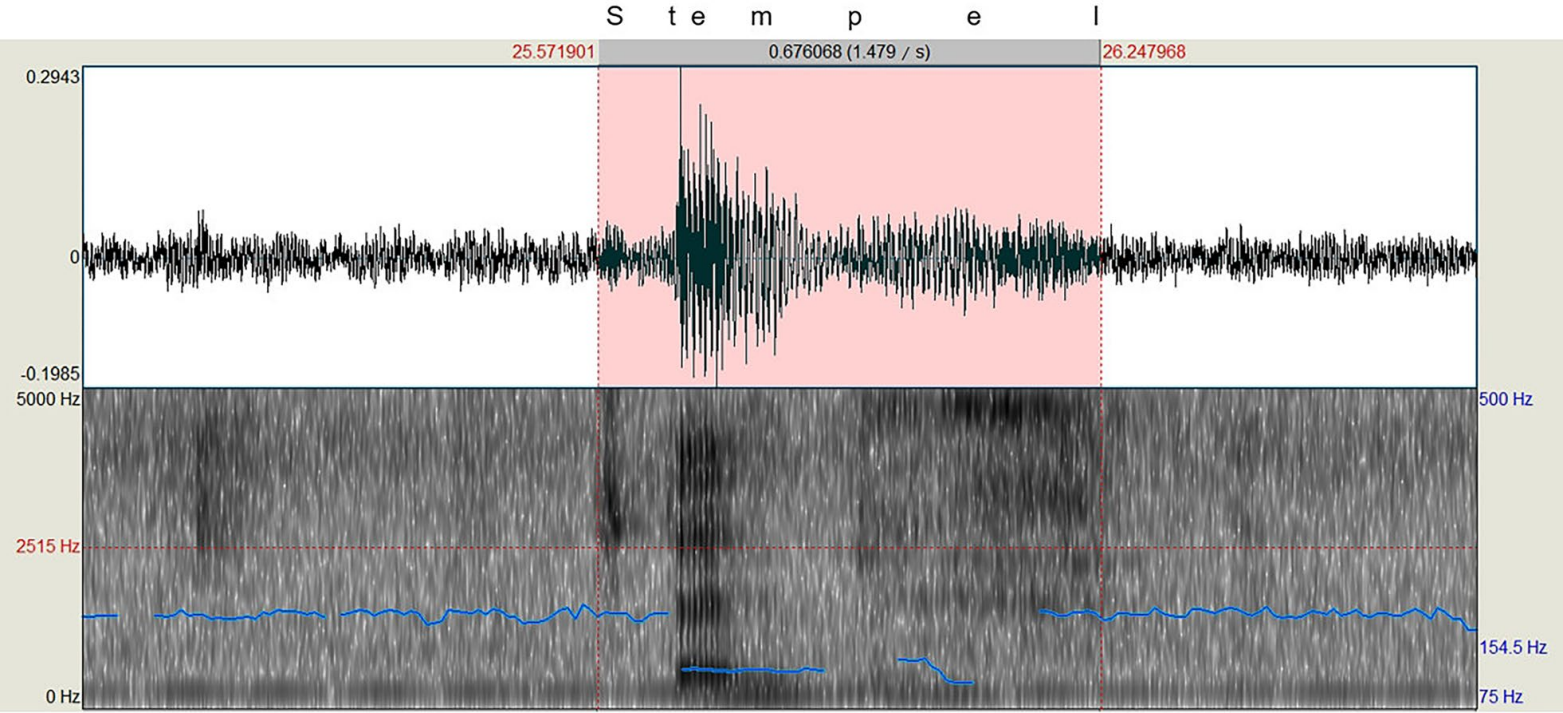
Measurement of voice-onset latencies. Pictured is the Praat interface, loaded with an audio file extracted from a object-naming task video. The specific named object was added above post hoc. Praat shows both the waveform of the audio data as well as a Fourier-Transformation, visualizing the formants

We used the built-in report mechanism of the NexSpeech module to get information on when each single trial began (trial start time). The recorded video files of .asf data type were copied to an external computer, where an in-house Matlab script was used that first separated the audio track from the video and then saved each audio track in the form of a .wav file. Subsequently, we performed RT measurements on the audio tracks using Praat (version 6.0.28; http://www.fon.hum.uva.nl/praat/; Fig. 1).

The respective response to each object was documented for each trial, and both voice onset (time at which the response to a given trial began) and voice offset (time at which the response was finished) were measured and noted. This allowed for immediate calculation of both WD and RT by subtraction of voice onset from voice offset (for WD) and subtraction of trial start time from voice onset (for RT), respectively.

Different categories of possible errors were defined to characterize incorrect responses. There was a no response (NR) when the participant did not give any response or audibly indicated not knowing a proper response (e.g., “ehh…”), a performance error (PE) if the word was articulated in a flawed way such as by distorted pronunciation or through the interjection of inappropriate utterances (e.g., “tea-ehhh-pot”), and a semantic error (SE) if the given response was correctly articulated, but from a semantic point of view not adequate to describe the pictured object (e.g., “tomato” as a response to a picture of an orange) [27, 29–31].

### Data analyses

#### Statistical method

We performed confirmatory forward mixed-effects multiple regression analysis on the RTs of the L1 and L2. We used a mixed-effects multiple regression analysis, a method introduced into RT analyses of psycholinguistic studies to overcome problems regarding factorial study designs [26]. Mixed-effects multiple regression allows (1) to analyze all observations without averaging, (2) to test multiple, possibly interacting nominal and continuous factors, and (3) to estimate the genuine effect of each factor by partialling out the information common between fixed-effects factors and the random effects of participants and objects. In other words, mixed-effects regression allows to partial out the idiosyncrasies that participants and objects brought with them into the object-naming datasets in one model. Moreover, if the by-participant random intercept and the by-picture random intercept are significant, it means that the studied sample is diverse enough in terms of participants and objects. The significant diversity of the sample data, in turn, allows to generalize the results of the significant fixed-effects factors beyond the sample of participants and objects used in the present study.

Our approach is confirmatory in the sense that we tested preselected factors known to influence the RT according to previous studies as aforementioned. However, past studies have not shown the individual degree to which each factor accounts for the variance in its corresponding level of word production. Therefore, we performed a forward step-wise model comparison instead of a hierarchical model comparison.

#### Factors

Regarding the random effects, we tested the by-participant random intercept and the by-picture random intercept. Regarding the fixed-effects factors, we tested five types of variables. These variables include factors related to cognitive states (practice effect and/or fatigue effect) that would change over time (run numbers 1 vs. 2; trial numbers in each run from 1 to maximum 131), a factor related to language status (L1 vs. L2), factors related to semantic/lemma selection (test language run 1 percent correct [L1: 0.73–0.93; L2: 0.65–0.94]; task-relevant German run 1 percent correct [0.65–0.95]; WC: modal word vs. others), factors related to phonological code retrieval (log10 WF; first phoneme difference: same vs. different), factors related to articulatory load such as WD for included objects (ranging from 159 ms for “bi” in L1 Chinese [”fountain pen”] to 2165 ms for “panchina” in L2 Italian [”bench”]) and WD difference (L2–L1: ranging from − 1520 ms for “Mais” in L2 German [“corn on the cob”] to 1924 ms for “Trommel”, a non-modal name in L2 German for “Fass” [“barrel”]), as well as other demographic factors (age: 19 to 27 years; age of L2 acquisition: 0 to 10 years; gender: female vs. male). For the grouping factors, the slope was calculated as the change from the subgroup listed first to the subgroup listed second.

Regarding the fixed-effects interactions, we tested four interaction effects motivated by the current literature: language status × log10 WF interaction, age × log10 WF interaction, gender × first phoneme difference, and run number × trial number interaction. The factors of each interaction term are ordered so that the coefficient estimated for the interaction term is used to adjust the coefficient of the second factor for the first factor’s second subgroup. The information about the log10 WF for the object target names for the picture set used in the present study was taken from the SUBTLEX-DE [32].

Because our ultimate goal was to identify the contexts in which longer RTs are likely to occur during the object-naming task, instead of removing outlying longer RTs, the positively-skewed RT distribution was corrected by inverse-transforming the RT. Moreover, because WD and WD difference were also positively skewed, they were log10 transformed.

#### Local purposes

We planned two analyses for different foci. Analysis 1 was intended to compare the RTs of L1 and L2, with special attention to the first phoneme difference factor (same vs. different) and the WD difference factor. Analysis 2 was intended to compare only German RTs, with special attention to the WC factor (modal word vs. others) in addition to the first phoneme difference factor and the WD difference factor.

#### Data selection

We took three steps to select trials from the baseline datasets. First, we selected trials for which verbal responses were made in the 2500 ms time window and for which we were able to measure the RT properly. For Analysis 1, we paired up L1 and L2 trials for each object in each run of each participant, enabling us to calculate WD differences for each pair. We further grouped the paired words into one set in which the L1 and L2 translation did share the same first phoneme and one set in which they did not (3506 trials). Then, for Analysis 2, we selected German trials (1448 trials) from the trials selected for Analysis 1 and divided them according to whether or not the specific response was a modal or non-modal response.

#### Statistical procedures

Prior to the regression analysis, we corrected the positive skewness of the RT distribution by inverse transformation, in addition to log10 transformation of WD and WD difference mentioned earlier. We performed a forward model comparison, selecting at each step the factor that reduced the variance most among the factors that independently significantly reduced the variability in object-naming RT, with the threshold set at 0.05 for alpha.

For the forward model comparison, the empty model with only the fixed intercept was calculated first. Against this empty model, by-participant random intercept was tested. Next, the by-picture random intercept was tested. Then, the preselected fixed effects factors were tested one by one. Afterwards, the by-participant random slopes for fixed-effects factors and by-picture random slopes for fixed-effects factors were tested. Then, the two-way interaction effects between fixed factors were tested. The final model was rerun by using the restricted maximum likelihood method to obtain the unbiased variance components. In the final model, the order of factors in the regression equation was rearranged so that the analysis program forms the interaction terms consistent with the interaction hypotheses of the fixed effects. When a theoretically motivated 2-way interaction was significant, a-theoretic 3-way interactions were additionally tested to see if there was a significant 3-way interaction that would make the 2-way interaction non-significant and reduce the remaining variance significantly. It was also used to help localize the source of the effect of interest.

The assumptions for multiple regressions were examined for each final model, following Baayen [33]. To see if the residuals are normally distributed, standardized residuals were calculated and a density plot was generated for visual inspection. The skewness of the distribution was calculated to see if it would fall in the normal range between − 0.5 and + 0.5. To check the homoscedasticity assumption by visual inspection, fitted values are plotted along the horizontal axis and the corresponding standardized residuals were plotted along the vertical axis with the reference lines drawn at ± 2.5 for the standardized residuals. Trials with residuals that fell outside the ± 2.5 standard deviation (SD) were identified and tagged with actual reaction times and participants in order to find where in the range of reaction times the deviated residuals lay and see if they exclusively belonged to one or two participants.

For the final mixed-effects model, because there is no agreed-upon way of determining the degrees of freedom to translate the obtained t-values for the coefficient of each factor into p-values, p-values based on degrees of freedom returned by statistical programs may be misleading [34, 35]. Therefore, to complement the information, we provide the bootstrap confidence intervals (CIs) of each factor’s coefficient obtained by 10,000 times of bootstrapping in addition to providing the p-values determined by using the degrees of freedom calculated by Kenward and Roger’s method [36–38]. Additionally, the proportion of variance accounted for was calculated for the final model, the fixed effects, and the random effects.

In the results section, we report means and CIs of the back-transformed fitted RTs indicated by the subscript btf. To perform this series of statistical analysis, we used R (version 3.1.1; The R Foundation for Statistical Computing, Vienna, Austria) in combination with the lme4 package, the nlme package, lmerTest, krbttest, the MuMIn package, and the effects package [34, 36, 37, 39–42].

#### Analyses extended with a larger more representative and gender-balanced sample

Irreproducibility of results is a recently surging concern in neurobiology of language. The male sample (n = 3, contributing 561 trials) may not be representative to claim the gender effect and/or the first phoneme difference × gender effect even if 10,000-times bootstrap replications confirmed them. To address this concern, additional data were collected to see if the results of the first sample could be replicated with a larger, more representative, and more gender-balanced sample (n_female_ = 10, n_male_ = 10, in 7145 trials in total). With the time constraints imposed on the study 2 completion, the data were collected with a simplified procedure, scheduling the L1 and L2 sessions on the same day without the nTMS-related steps. In addition, the two samples differ in gender composite (7:3 vs. 3:7). Here, our report focuses on the replicability test of the effects detected in the sample that may be less representative and gender-imbalanced. At the end of the result section, a brief report was added to mention two of the interactions that were part of the decomposition of sample difference and relevant to the present-theory testing investigation.

The data from the previous analysis was combined with the new data set. Using this larger, more representative, and gender-balanced data set, the final models of analysis 1 and analysis 2 were tested. Where applicable, the hypothesized effects that were not significant in sample 1 were added to the final model to see if they would become significant with a larger, more representative, gender-balanced sample. These hypotheses included word frequency × age (or age of L2 acquisition) for the Weaker Links hypothesis from Analysis 1 and word frequency × word choice interaction for the Cascade model from analysis 2. Because the first replicability test asks if there are non-contributing terms in the proposed final model, backward model comparisons for elimination was performed instead of forward model comparison. To be consistent, subsequent testing of the previously non-significant terms was also performed by backward model comparison. The threshold for elimination was set at α = 0.05. As the model increases its complexity with the doubled sample size, calculating the Kenward and Roger degrees of freedom became impractically time-consuming. The default method of calculating the degrees of freedom (Satterthwaite method) was used. The bootstrap test was performed with 10,000 replications as was done in the previous analyses. When the effects package did not generate the plot to show the specific aspect of the interaction between a continuous variable and a categorical variable or between continuous variables, the fitted means and confidence intervals were calculated in the effect package and the result was reorganized and plotted by our custom scripts.

## Results

### Analysis 1: Analysis including L1 vs. L2 comparisons

#### Analysis 1: Overview

3506 trials from 10 participants in responses to 131 objects were analyzed. As shown by the model comparison (Table 2), the forward-model comparisons arrived at the final model that consisted of the by-participant random intercept, the by-picture random intercept, five fixed-effects factors (run number, trial number, first phoneme difference, language status, and log10 WF), and three two-way interactions (run number × log10 WF, language status × log10 WF, and run number × trial number). The final model accounted for 34.91% of the variance. The by-participant random intercept and the by-picture random intercept jointly accounted for 22.38% of the variance. The five fixed-effects terms and the three interaction terms jointly accounted for 12.53% of the variance. The variables related to the articulatory effort were not contributing factors.

**Table 2:** Analysis 1 (L1 and L2 combined): model comparison

Models	Information criteria (log likelihood)	Deviance (− 2* log likelihood)	Number of parameters	Chi-square obtained	df	p value
Fixed EffectI only	25,324.67	− 50,625.74	2			
Plus subjI	25,544.43	− 51,088.86	3	439.51	1	p < 0.0001
Plus subjI. ItemI	25,707.13	− 51,414.26	4	325.41	1	p < 2.2e−16
Plus runNum	25,386.19	− 51,672.38	5	258.11	1	p < 2.2e−16
Plus trialNum	25,879.00	− 51,758.00	6	85.62	1	p < 2.2e−16
Plus firstPhonemeDiff	25,903.81	− 51,807.62	7	49.62	1	p = 1.865e−12
Plus langStatus	25,917.20	− 51,834.40	8	26.78	1	p = 1.827e−06
Plus log10WF	25,928.58	− 51,857.16	9	22.77	1	p = 0.0001816
Plus runNum*log10WF	25,935.73	− 51,871.46	10	14.29	1	p = 0.0001564
Plus langStatus*log10WF	25,942.74	− 51,885.48	11	14.01	1	p = 0.0001816
Plus runNum*trialNum	25,947.10	− 51,894.20	12	8.73	1	p = 0.003138

This table provides a comparison of different statistical models used for the reaction time (RT) comparisons between the first language (L1) and second language (L2)

**Fig. 2:**
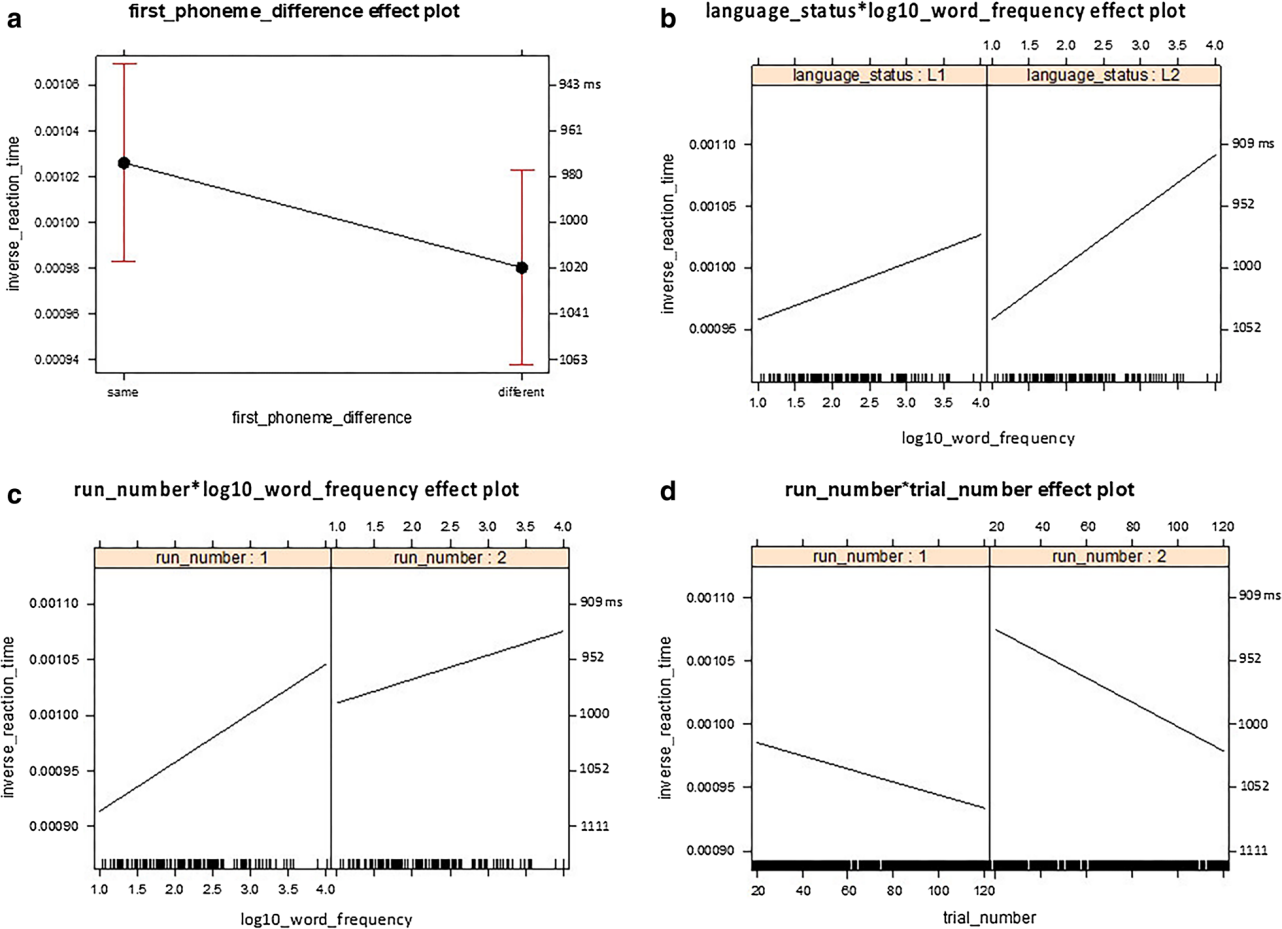
Analysis 1: Inter-language comparisons. This figure illustrates the means and confidence intervals (CIs) of the fitted inverse reaction time (RT) for the fixed-effects factors and the interaction terms visible in **a**–**d** with the right vertical axis annotated with back-transformed reaction times in ms. RT is shorter as it is higher up along the vertical axis

For the verbal summary about the continuous variable factors (log10 WF and trial numbers), means and CIs of the RTs are represented at log10 WF = 1 (10 occurrences per million) as low frequency, log10 WF = 4 (10,000 occurrences per million) as high frequency, trial number 20th as earlier trials and trial number 120th as later trials. For an overview, see Tables 2, 3 and 4 and Fig. 2.

**Table 3:** Analysis 1 (L1 and L2 combined): final model fixed effects

Terms	Estimate	Std. error	T-obt	95% CI lower	95% CI upper	K&R df	p-value	Sign.
Intercept	9.409E−04	2.840E−05	33.125	8.868E−04	9.961E−04	29	< 2e−16	***
Run Num (run 2)	1.467E−04	1.631E−05	8.991	1.137E−04	1.781E−04	3405	< 2e−16	***
Trial_number	− 5.155E−07	8.845E−08	− 5.827	− 6.879E−07	− 3.442E−07	3425	6.17E−09	***
First PhonemeDiff (diff)	− 4.571E−05	6.413E−06	− 7.113	− 5.825E−05	−3.313E−05	3399	1.37E−12	***
langStatus (L2)	− 2.117E−05	1.342E−05	− 1.578	− 4.781E−05	5.302E−06	3361	0.1147	
log10WF	3.331E−05	7.882E−06	4.225	1.762E−05	4.897E−05	216	3.52E−05	***
langStatus (L2)*log10WF	2.148E−05	5.818E−06	3.692	1.005E−05	3.284E−05	3361	2.26E−04	***
Run Num (run 2)*log10WF	− 2.257E−05	5.883E−06	− 3.837	− 3.401E−05	− 1.104E−05	3389	1.27E−04	***
Run Num (run 2)*trialNum	− 4.414E−07	1.495E−07	− 2.951	− 7.249E−07	−1.403E−07	3418	0.0032	**

This table provides an overview reflecting the final statistical model used for the comparison of the first language (L1) and second language (L2). In the table, the name of the subgroup in parentheses is the subgroup to which the regression slope is calculated as the change from the other subgroup. A pair of factors of an interaction term is ordered in a way that the coefficient estimated for the interaction term is used to adjust the coefficient of the second factor for the first factor’s second subgroup

**Table 4:** Analysis 1 (L1 and L2 combined): back-transformed fitted reaction times (ms)

Fixed-effect levels	Fitted mean	95% CI lower bound	95% CI upper bound
firstPhoneme_Same	974	935	1017
firstPhoneme_Different	1020	877	1066
L1, WF = 10/Mil.	1044	996	1096
L1, WF = 10,000/Mil.	973	927	1024
L2, WF = 10/Mil.	1043	996	1095
L2, WF = 10,000/Mil.	916	875	960
Run 1, WF = 10/Mil.	1094	1042	1151
Run 1, WF = 10,000/Mil.	956	912	1004
Run 2, WF = 10/Mil.	989	946	1036
Run 2, WF = 10,000/Mil.	929	887	976
Run 1, Trial number 20th	1014	972	1061
Run 1, Trial number 120th	1070	1023	1123
Run 2, Trial number 20th	930	894	969
Run 2, Trial number 120th	1021	976	1071

This table illustrates the condition-specific reaction time (RT) means with upper and lower 95% confidence interval (CI) bounds as related to their respective analysis groups

The skewness of the distribution of the residuals fell in the range of normal distribution (skewness − 0.49). Homoscedasticity assumption was not violated by visual inspection. Residuals outside 2.5 SD occupied 1.96% of the trials (69 out of 3506) and all participants in the analysis contributed 3–14 trials (median = 5.5). The 10,000 times bootstrap test showed that all the significant factors and interactions were stable.

#### Analysis 1: Random effects

Regarding the random effects, adding the by-participant random intercept first (*χ*^*2*^(1) = 439.51, *p* < 0.0001) and adding the by-picture random intercept second (*χ*^*2*^(1) = 325.41, *p* < 2.2e−16) both significantly reduced the variance (Table 2). These results suggest that for the final model reported, by partialling out the idiosyncrasies of the participants and the objects in the sample, significant effects of the fixed-effects factors and their interactions are generalizable beyond the participants and the objects employed in the present study. Regarding the by-participant random intercept (*SD* = 6.603e−05, 95% CI 3.590e−05, 9.617e−05), the back-transformed adjusted random intercepts ranged from 936 to 1162 ms. Regarding the by-picture random intercept (*SD *= 5.566e−05, 95% CI 4.678e−05, 6.414e−05), the back-transformed adjusted random intercepts ranged from 938 ms for “Schlange” (”snake”) to 1211 ms for “Kommode” (”dresser”).

#### Analysis 1: Fixed effects

##### Fixed-effects factors related to phonological code retrieval

*First phoneme difference* The first-phoneme difference factor was significant (*b* = − 4.571e−05, *t*(3399) = − 7.113, *p* = 1.37e−12) and did not interact with other factors (Table 3). More specifically, RT was, on average, shorter for the trials with the same first phoneme (*M*_btf_ = 974 ms, 95% CI_btf_ 935 ms, 1017 ms) than for the trials with the different phonemes (*M*_btf_ = 1020 ms, 95% CI_btf_ 977 ms, 1066 ms; Fig. 2a, Table 4).

*Language status × log10 word frequency degree interaction* The log10 WF factor was significant (*b* = 3.331e−05, *t*(216) = 4.225, *p* = 3.52e−05) but more important, there was a significant degree interaction effect between the language status factor and the log10 WF factor (*b*_interaction_ = 2.148e−05, *t*(3361) = 3.692, *p* = 2.26e−04; Table 3). Due to this interaction, although adding the language status factor significantly reduced the variance earlier in the forward model comparison (*χ*^*2*^(1) = 26.78, *p* = 1.827e−06; Table 2), the coefficient of the language status factor was non-significant in the final model (*b* = − 2.117e−05, *t*(3361)= − 1.578, *p* = 0.1147; Table 3).

More specifically, RTs were, on average, shorter for the high-frequency words (log10 WF = 4) than for the low-frequency words (log10 WF = 1), but the difference between the high-frequency words and the low-frequency words was greater for L2 (L2 high: *M*_btf_ = 916 ms, 95% CI_btf_ 875 ms, 960 ms; L2 low: *M*_btf_ = 1043 ms, 95% CI_btf_ 996 ms, 1095 ms) than for L1 (L1 high: *M*_btf_ = 973 ms, 95% CI_btf_ 927 ms, 1024 ms; L1: low M_btf_ = 1044 ms, 95% CI_btf_ 996 ms, 1096 ms) and the L2 high-frequency words received the shortest RTs (Fig. 2b, Table 4). The RTs were, on average, shorter during L2 object naming than during the L1 object naming in the present sample. Now even though the L2 may thus be the language of currently dominant use, the hypothesis that the difference between the high-frequency words and the low-frequency words being greater in the L2 than in the L1 nevertheless correctly distinguished the L2 from the L1 in the present sample.

None of the additional a-theoretical 3-way interactions (language status × word frequency × run number, or × trial number, or × first phoneme difference) were significant, made the significant two-way interaction non-significant, or significantly reduced the variance at the same time.

##### Factors related to cognitive states

*Run number* × *log10 word frequency degree interaction* There was a significant effect of the run-number factor (*b* = 1.467e−04, *t*(3405)= 8.991, *p* < 2e−16) in addition to the significant effect of the log10 WF factor reported earlier. More importantly, there was a significant degree interaction effect between the run number factor and the log10 WF factor (*b*_interaction_ = − 2.257e−05, *t*(3389)= − 3.837, *p* = 1.27e−04; Table 3). More specifically, RT was, on average, shorter for the high-frequency words than for the low-frequency words. Besides, RT was shorter in run 2 than in run 1, which suggests a practice effect. Furthermore, the RT difference between the high-frequency words and the low-frequency words was smaller in run 2 (Run 2 high: *M*_btf_ = 929 ms, 95% CI_btf_ 887 ms, 976 ms; Run 2 low: *M*_btf_ = 989 ms, 95% CI_btf_ 946 ms, 1036 ms) than in run 1 (Run 1 high: *M*_btf_ = 956 ms, 95% CI_btf_ 912 ms, 1004 ms; Run 1 low: *M*_btf_ = 1094 ms, 95% CI_btf_ 1042 ms, 1151 ms), possibly due to fatigue effects depriving the high-frequency words of their advantage (Fig. 2c, Table 4).

None of the additional a-theoretical 3-way interactions (run number × word frequency × trial number, or × first phoneme difference, or × language status) were significant, made the significant two-way interaction non-significant, or significantly reduced the variance at the same time.

*Run number* × *trial number degree interaction* There was a significant effect of the run-number factor and a significant effect of the trial-number factor (*b* = − 5.155e−07, *t*(3425) = − 5.827, *p* = 6.17e−09). More important, there was a significant degree interaction effect between the run-number factor and the trial-number factor (*b*_interaction_ = − 4.414e−07, *t*(3418)= − 2.951, *p* = 0.0032, Table 3). More specifically, RT was, on average, shorter in run 2 than in run 1, suggesting a practice effect. Also, RT was shorter for the earlier trials than for the later trials, suggesting a fatigue effect developing over 131 trials. Furthermore, the RT difference between the earlier trials and the later trials was greater for run 2 (Run 2 20th trial: *M*_btf_ = 930 ms, 95% CI_btf_ 894 ms, 969 ms; Run 2 120th trial: *M*_btf_ = 1021 ms, 95% CI_btf_ 976 ms, 1071 ms) than for run 1 (Run 1 20th trial: *M*_btf_ = 1014 ms, 95% CI_btf_ 927 ms, 1061 ms; Run 1 120th trial: *M*_btf_ = 1070 ms, 95% CI_btf_ 1023 ms, 1123 ms) depriving the later trials in run 2 of the practice effect advantage (Fig. 2d, Table 4).

None of the additional a-theoretical 3-way interactions (run number × trial number × first phoneme difference, or × language status, or × word frequency) were significant, made the significant two-way interaction non-significant, or significantly reduced the variance at the same time.

### Analysis 2: German object naming only

#### Analysis 2: Overview

1448 trials from eight participants in responses to 131 objects were analyzed. The forward-model comparisons arrived at the final model that consisted of the by-participant random intercept, the by-picture random intercept, seven fixed-effects factors (run number, trial number, log10 WF, first phoneme difference, WC, German run 1 percent correct, and participant’s gender) and two two-way interactions (gender × first phoneme difference, German run 1 percent correct × log10 WF).

The skewness of the distribution of the residuals fell in the range of normal distribution (skewness − 0.49). Homoscedasticity assumption was not violated by visual inspection. Residuals outside 2.5 SD occupied 2.14% of the trials (31 out of 1448) and all participants in the analysis contributed 1–9 trials (median = 3.5). The 10,000 times bootstrap test showed that all the significant factors and interactions were stable.

**Fig. 3:**
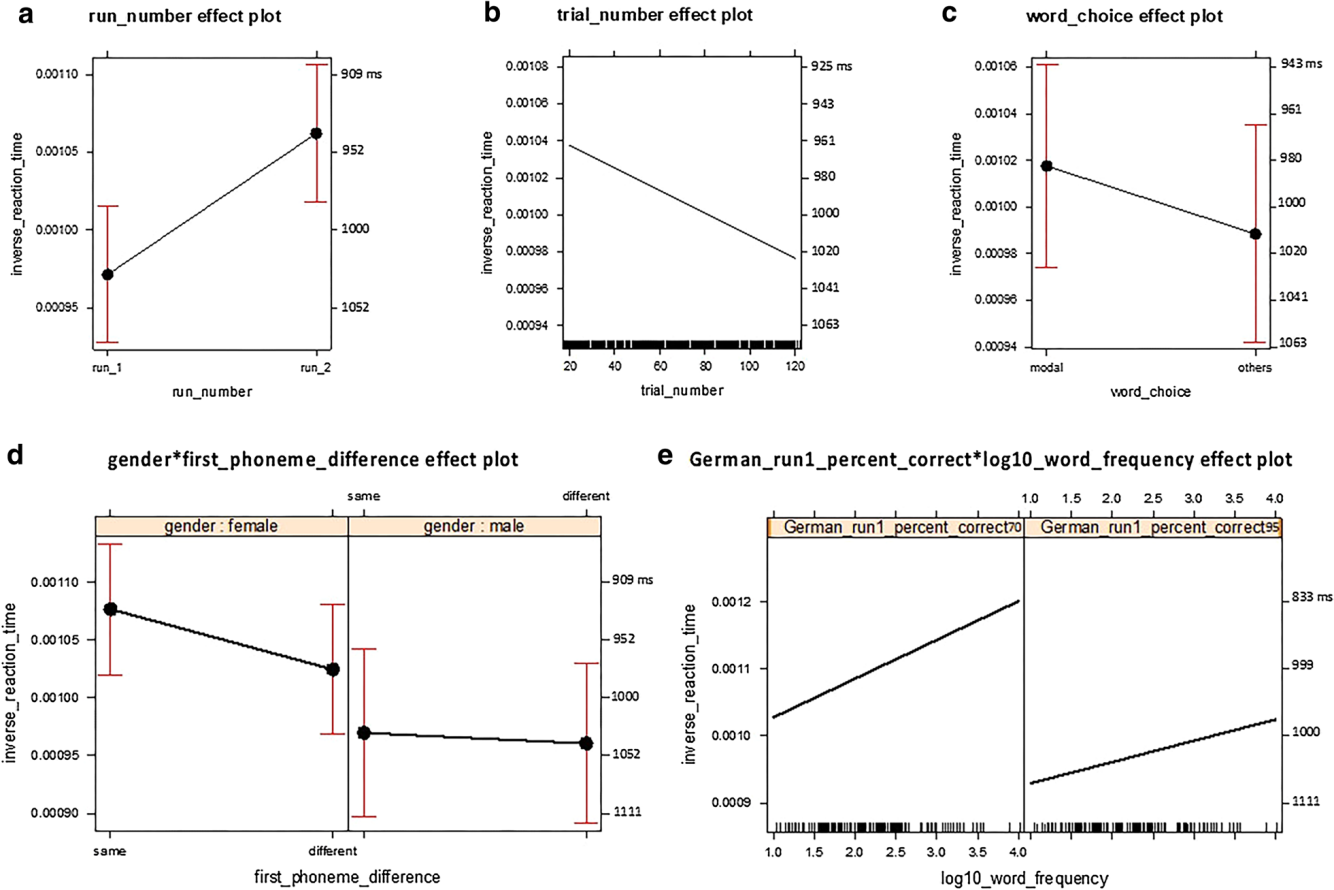
Analysis 2: Intra-language comparisons for German production. This figure visualizes the means and confidence intervals (CIs) of the fitted inverse reaction time (RT) for the fixed-effects factors and the interaction terms visible in **a**–**e** with the right vertical axis annotated with back-transformed reaction times in ms. RT is shorter as it is higher up along the vertical axis

The final model accounted for 48.41% of the variance. More specifically, the by-participant intercept and the by-picture intercept jointly accounted for 20.99% of the variance, while the seven fixed-effects terms and the two fixed-effects interaction terms jointly accounted for 27.42% of the variance. The variables related to the articulatory effort were not contributing factors. For an overview, see Tables 5, 6, 7 and Fig. 3.

**Table 5:** Analysis 2 (German only): model comparison

Models	Information criteria (log likelihood)	Deviance (− 2* log likelihood)	Number of parameters	Chi-square obtained	df	p-value
Fixed EffectI only	10,363.62	− 20,704.64	2			
Plus subjI	10,509.75	− 21,019.50	3	292.27	1	p < 0.0001
Plus subjI. ItemI	10,589.46	− 21,178.92	4	159.41	1	p < 2.2e−16
Plus runNum	10,671.27	− 21,342.54	5	163.63	1	p < 2.2e−16
Plus trialNum	10,686.06	− 21,372.12	6	29.56	1	p = 5.416e−08
Plus log10WF	10,697.36	− 21,394.72	7	22.61	1	p = 1.986e−05
Plus firstPhonemeDiff	10,704.05	− 21,408.10	8	13.39	1	p = 0.000253
Plus wordChoice	10,707.79	− 21,415.58	9	7.46	1	p = 0.006300
Plus GermanRun1PercentCorrect	10,710.99	− 21,421.98	10	6.40	1	p = 0.011383
Plus gender	10,713.03	− 21,426.06	11	4.09	1	p = 0.043243
Plus gender*firstPhonemeDiff	10,715.07	− 21,430.14	12	4.08	1	p = 0.043447
Plus GermanRun1PercentCorrect*log10WF	10,717.46	− 21,434.92	13	4.78	1	p = 0.028846

This table shows a comparison of different statistical models used for the within-German reaction time (RT) comparisons

**Table 6:** Analysis 2 (German only): final model fixed effects

Terms	Estimate	Std. Error	T-obt	95% CI lower	95% CI upper	K&R df	p-value	Sign.
Intercept	1.218E−03	2.459E−04	4.950	8.269E−04	1.788E−03	8.4	9.77E−04	***
Run Num (run 2)	9.064E−05	7.519E−06	12.053	7.603E−05	1.055E−04	1329	< 2e−16	***
trial_number	− 6.108E−07	1.091E−07	− 5.598	− 8.212E−07	− 3.916E−07	1376	2.61E+08	***
GermanRun1%Correct	− 3.631−04	2.836E−04	−1.280	−9.200E−04	1.901E−04	8.1	0.2359	
wordChoice (others)	− 2.916E−05	1.083E−05	− 2.688	− 5.039E−05	−8.429E−06	1434	0.0073	**
Gender	−1.068E−04	4.645E−05	− 2.298	− 1.970E−04	− 1.540E−05	5.9	0.0624	
log10WF	1.548E−04	5.266E−05	2.938	5.285E−05	2.563E−04	1402	0.0034	**
firstPhonemeDiff	− 5.199E−05	1.208E−05	− 4.281	− 7.596E−05	− 2.777E−05	1410	1.98E−05	***
gender*firstPhonemeDiff	4.277E−05	1.853E−05	2.302	6.279E−06	7.984E−05	1388	0.0215	*
GermanRun1%Correct*log10WF	− 1.295E−04	5.904E−05	− 2.193	−2.437E−04	−1.494E−05	1346	0.0285	*

This table provides an overview reflecting the final statistical model used for the within-German reaction time (RT) comparisons. The degrees of freedom to determine the p-values were calculated using Kenward and Roger’s method. The p-value indicates that the gender factor was only marginally significant. However, the 10,000 bootstrap CI that did not include 0 suggests that the gender factor was reliable

**Table 7:** Analysis 2 (German only): back-transformed fitted reaction times (ms)

Fixed-effect levels	Fitted mean	95% CI lower bound	95% CI upper bound
runNum run1	1029	985	1078
runNum run2	942	904	982
trialNum 20th	964	924	1007
trialNum 120th	1024	979	1074
wordChoice modal	983	942	1027
wordChoice others	1012	966	1062
Female, firstPhoneme_Same	929	882	981
Female, firstPhoneme_Diff	976	926	1032
Male, firstPhoneme_Same	1031	959	1115
Male, firstPhoneme_Diff	1041	971	1122
GermanRun1 = 70% correct, WF = 10/Mil.	950	869	1049
GermanRun1 = 70% correct, WF = 10,000/Mil.	803	741	878
GermanRun1 = 95% correct, WF = 10/Mil.	1076	1008	1154
GermanRun1 = 95% correct, WF = 10,000/Mil.	976	915	1047

This table displays the reaction time (RT) means with upper and lower 95% confidence interval (CI) bounds as related to their respective analysis groups

#### Analysis 2: Random effects

Regarding the random effects, adding the by-participant random intercept first (*χ*^*2*^(1) = 292.27, *p* < 0.0001) and adding the by-picture random intercept second (*χ*^*2*^(1) = 159.41, *p* < 2.2e−16) both significantly reduced the variance (Table 5). These results suggest that, for the final model reported below, by partialling out the idiosyncrasies of the participants and the objects in the sample, significant effects of the fixed-effects factors and their interactions are generalizable beyond the participants and the objects employed in the present study. Regarding the by-participant random intercept (*SD* = 5.878e−05, 95% CI 2.143e−05, 9.512e−05), the back-transformed adjusted intercepts ranged from 722 ms to 811 ms. Regarding the by-picture random intercept (*SD *= 6.634e−05, 95% CI 5.418e−05, 7.790e−05), the back-transformed adjusted intercepts ranged from 692 ms for “Schreibtischstuhl” (”desk chair”) to 858 ms for “Kamera” (”camera”).

#### Analysis 2: Fixed effects

##### Fixed-effects factors related to semantic or lemma selection

*Word choice* The word-choice factor was significant (*b* = − 2.916E−05, *t*(1434) = − 2.688, *p* = 0.0073) and did not interact with other factors. More specifically, RT was, on average, longer for the naming responses of non-modal words (*M*_btf_ = 1012 ms, 95% CI_btf_ 966–1062 ms) than for the naming responses of modal words (*M*_btf_ = 983 ms, 95% CI_btf_ 942–1027 ms), regardless of other factors (Fig. 3c).

*German run 1 percent correct* × *log10 word frequency degree interaction* There was a significant degree interaction (*b*_interaction_ = − 1.295e−04, *t*(1342) = − 2.193, *p* = 0.0285) between the German run 1 percent correct factor and a factor related to phonological code retrieval (log10 WF) (*b*_log10WF_ = − 1.548e−04, *t*(1402) = 2.938, *p* = 0.0034). Due to this interaction, although the German run 1 percent correct factor significantly reduced the variance earlier in the forward model comparison (*χ*^*2*^(1) = 6.40, *p* = 0.011383, Table 5), the coefficient of the German run 1 percent correct factor was non-significant in the final model (*b* = − 3.631e−04, *t*(8.1) = − 1.280, *p* = 0.2359, Table 6).

More specifically, RT was, on average, shorter for higher-frequency words than for lower-frequency words. Furthermore, the difference between the high-frequency words and the low-frequency words was greater for the participants with lower German run 1 percent correct (70% correct, high frequency: *M*_btf_ = 803 ms, 95% CI_btf_ 741 ms, 878 ms; 70% correct, low frequency: *M*_btf_ = 950 ms, 95% CI_btf_ 869 ms, 1049 ms) than for the participants with higher German run 1 percent correct (95% correct, high frequency: *M*_btf_ = 976 ms, 95% CI_btf_ 915 ms, 1047 ms; 95% correct, low frequency: *M*_btf_ = 1076 ms, 95% CI_btf_ 1008 ms, 1154 ms), with the advantage associated with higher frequency words attenuated for those high in German run 1 percent correct (Fig. 3e, Table 7).

None of the additional a-theoretical 3-way interactions (German run 1 percent correct × word frequency × run number, or × trial number, or × first phoneme difference, or × word choice, or × gender) were significant, made the significant two-way interaction non-significant, or significantly reduced the variance at the same time.

##### Factors related to phonological code retrieval

In addition to the log10 WF factor reported earlier, the first-phoneme difference factor was significant (*b* = − 5.199e−05, *t*(1410) = − 4.281, *p* = 1.98e−05). Moreover, there was a significant degree interaction between the gender factor and the first-phoneme difference factor (*b*_interaction_ = 4.277E−05, *t*(1388) = 2.302, *p* = 0.0215, Table 6). The RT was, on average, shorter for the trials of L1–L2 target words sharing the same first phoneme than for the trials in which L1–L2 target words started with different phonemes. More importantly, the RT difference between the trials of the L1–L2 target words starting with different first phonemes and the trials of the L1–L2 target words sharing the same first phoneme was smaller for male participants (male, first phoneme diff: *M*_btf_ = 1041 ms, 95% CI_btf_ 971 ms, 1122 ms; male, first phoneme same: *M*_btf_ = 1031 ms, 95% CI_btf_ 959 ms, 1115 ms) than for female participants (female, first phoneme diff: *M*_btf_ = 976 ms, 95% CI_btf_ 926 ms, 1032 ms; female, first phoneme same: *M*_btf_ = 929 ms, 95% CI_btf_ 882 ms, 981 ms, Fig. 3d, Table 7).

**Fig. 4:**
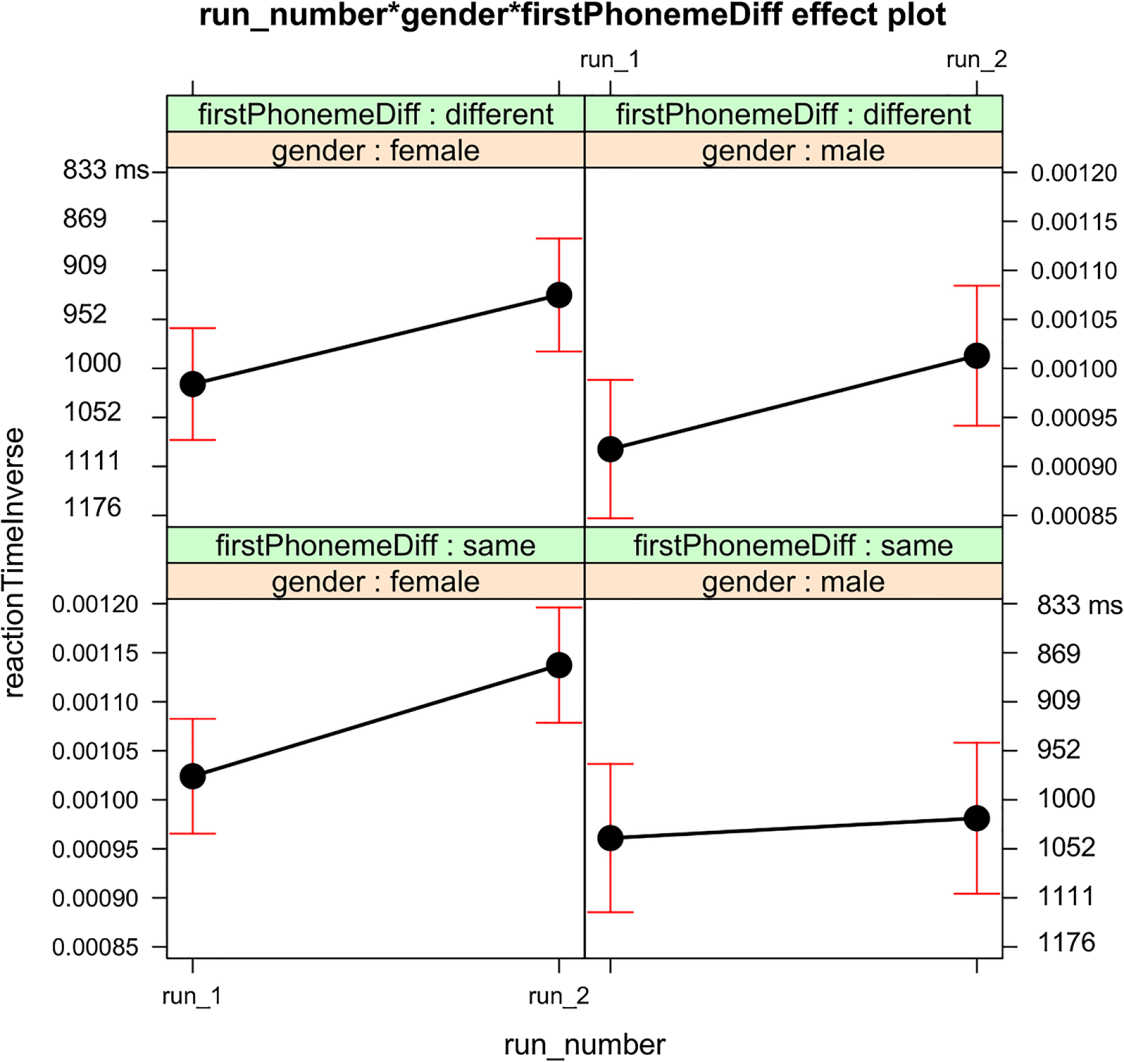
Analysis 2: A-theoretical three-way interaction. This figure visualizes the means and confidence intervals (CIs) of the fitted inverse reaction time (RT) for the a-theoretical three-way interaction of fixed-effects factors with the left top and right bottom vertical axes annotated with back-transformed reaction times in ms. RT is shorter as it is higher up along the vertical axis

Four of the additional a-theoretical 3-way interactions (first phoneme difference × gender × trial number, or × word frequency, or × word choice, or × German run 1 percent correct) were non-significant and did not significantly reduced the remaining variance. First phoneme difference × gender × run number was significant (p = 0.003) and significantly reduced the remaining variance jointly with the other two automatically added a-theoretical two-way interactions (p = 0.008). The theoretically motivated two-way interaction (first phoneme difference × gender) became non-significant, whereas one of the automatically added a-theoretic two-way interaction run number × gender was significant (p = 0.0006). The first phoneme factor remained significant with the benefit by the same first phonemes. These results together showed that the significant run number × gender interaction depended on the first phoneme difference factor (Fig. 4). Female participants benefitted from the second run regardless of the first phoneme difference factor. In contrast, male participants benefitted in the second run when the first phonemes were different, whereas they did not benefit from the second run when the first phonemes were the same. Therefore, the source of the lack of language-independent phonological activation in male participants was localized in this condition (Fig. 4, right bottom panel).

##### Factors related to cognitive states

*Run number* The run-number factor was significant (*b* = 9.064e−05, *t*(1329) = 12.053, *p* < 2e−16, Table 6) and did not interact with other factors. More specifically, RT was, on average, shorter in run 2 (*M*_btf_ = 942 ms, 95% CI_btf_ 904 ms, 982 ms) than in run 1 (*M*_btf_ = 1029 ms, 95% CI_btf_ 985 ms, 1078 ms), regardless of other factors (Fig. 3 panel a, Table 7). The result suggests a robust practice effect.

*Trial number* The trial-number factor was significant (*b* = − 6.108E−07, *t*(1376) = − 5.598, *p* < 2.61e−08, Table 6) and did not interact with other factors. More specifically, RT was, on average, longer for later trials (trial number 120th *M*_btf_ = 1024 ms, 95% CI_btf_ 979 ms, 1074 ms) than for earlier trials (trial number 20th *M*_btf_ = 964 ms, 95% CI_btf_ 924 ms, 1007 ms), regardless of other factors (Fig. 3b, Table 7). The result suggests a robust fatigue effect building up steadily during each run for the 5 min 30 s.

### Analysis 3 (Analysis 1 extended with n = 20)

#### Analysis 3 Overview

**Fig. 5:**
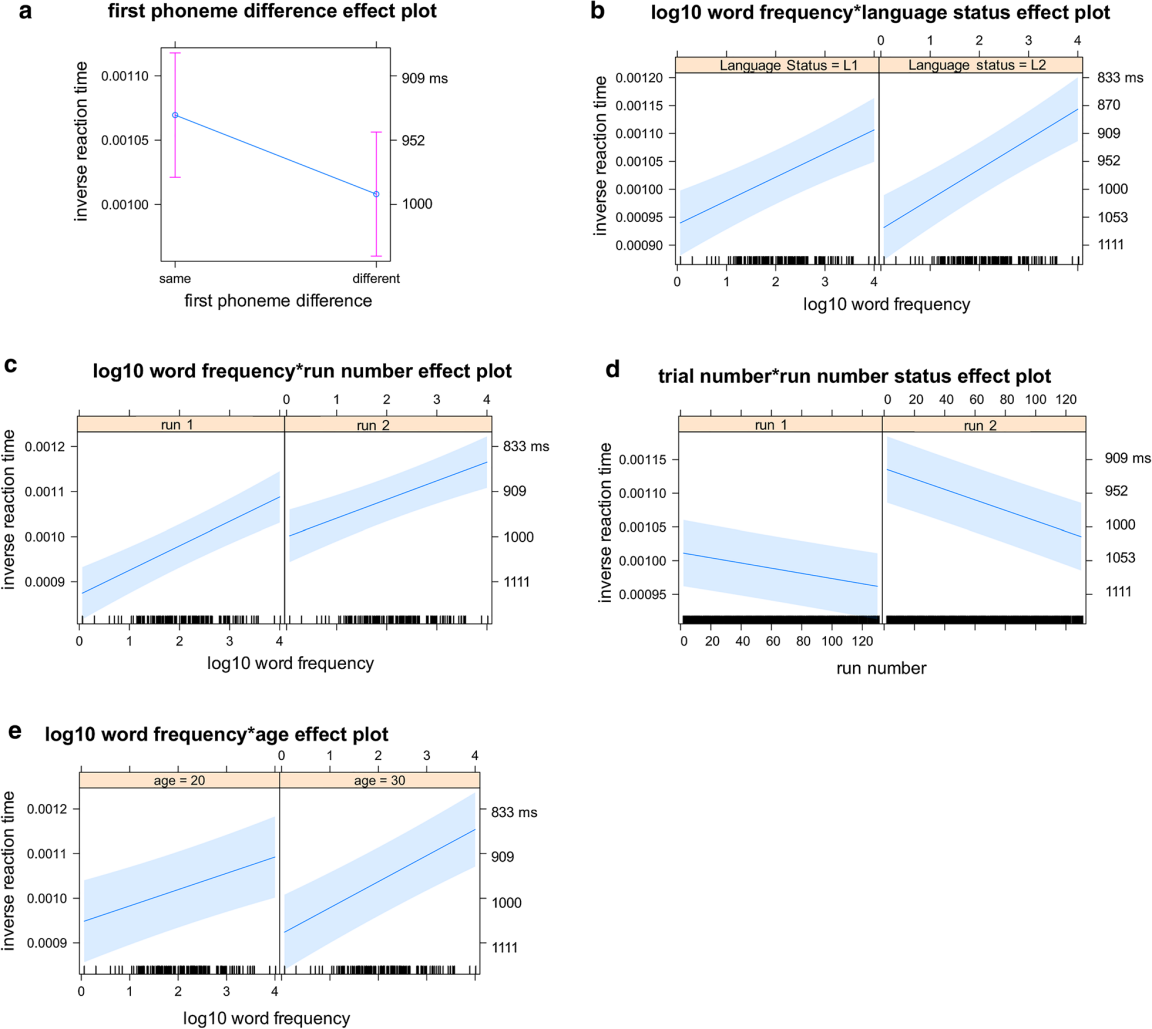
Interactions determined in analysis 3. This figure details findings made in analysis 3. This entails the influence of first phoneme difference (**a**), word frequency × language status (**b**), word frequency × run number (**c**), trial number × run number (**d**) and word frequency × age (**e**) on reaction time (RT)

7145 trials from 20 participants in responses to 131 objects were analyzed. The data set consisted of 3471 trials from 10 female participants and 3674 trials from 10 male participants, and thus, it was gender-balanced. The final model consisted of the fixed intercept, the by-participant random intercept, the by-picture random intercept, six fixed-effects factors and four 2-way interactions (Tables 8 and 9, Fig. 5). First phoneme difference, word frequency × language status, word frequency × run number, and trial number × run number were replicated. Word frequency × age became significant with this large sample. The 2-way interaction was predicted by the Weaker Links hypothesis. However, contrary to the prediction, the advantage of the higher frequency words over lower frequency words was greater for older participants than for the younger participants.

**Table 8:** Analysis 3 (n = 20, L1 and L2): final model by backward model comparison

Terms	Eliminated	npar	logLik	AIC	LRT	df	p value
Fixed intercept		14	51,279.51	− 102,531.00			
(1 | partID)	0	13	50,411.47	− 100,796.90	1736.074	1	~ 0.000
(1 | picID)	0	13	50,987.03	− 101,948.10	584.9555	1	3.135e−129

Terms	Eliminated	Sum Sq	Mean Sq	NumDF	DenDF	F value	p value
firstPhonemeDiff	0	3.992E−06	3.992E−06	1	7053.577	127.485	2.59E−29
log10WF:langStatus	0	1.552E−07	1.552E−07	1	6984.862	4.955	0.026
log10WF:runNum	0	1.889E−07	1.889E−07	1	7002.267	6.031	0.014
runNum:trialNum	0	3.151E−07	3.151E−07	1	7023.822	10.060	0.002
log10WF:age	0	2.622E−07	2.622E−07	1	7020.770	8.372	0.004

This table provides a comparison of different statistical models used for analysis 3

**Table 9:** Analysis 3 (n = 20, L1 and L2): final model table of coefficients

Terms	Estimate	Std. error	95% CI lower	95% CI upper	t value	df	p value
(Intercept)	9.982E−04	1.761E−04	6.546E−04	1.344E−03	5.667	20.714	1.330E−05***
firstPhonemeDiffdifferent	− 6.144E−05	5.442E−06	− 7.213E−05	− 5.076E−05	− 11.291	7053.577	2.593E−29***
log10WF	− 7.034E−06	2.078E−05	− 4.707E−05	3.380E−05	− 0.339	3770.405	0.735
langStatusL2	− 8.887E−06	1.189E−05	− 3.197E−05	1.419E−05	− 0.747	6984.939	0.455
runNum2	1.523E−04	1.414E−05	1.244E−04	1.802E−04	10.774	7015.487	7.412E−27***
trialNum	− 3.851E−07	7.789E−08	− 5.319E−07	− 2.337E−07	− 4.944	7025.205	7.831E−07***
Age	− 2.596E−06	6.835E−06	− 1.605E−05	1.092E−05	− 0.380	20.340	0.708
log10WF:langStatusL2	1.148E−05	5.157E−06	1.611E−06	2.149E−05	2.226	6984.862	0.026*
log10WF:runNum2	− 1.273E−05	5.184E−06	− 2.281E−05	− 2.510E−06	− 2.456	7002.267	0.014*
runNum2:trialNum	− 3.935E−07	1.241E−07	− 6.367E−07	− 1.507E−07	− 3.172	7023.822	0.002*
log10WF:age	2.193E−06	7.579E−07	6.665E−07	3.674E−06	2.893	7020.770	0.004*

This table details the influence of various coefficients for the statistical model used in analysis 3

The extended model accounted for 40.79% of the variance. The by-participant random intercept and the by-picture random intercept jointly accounted for 29.42% of the variance. The six simple fixed-effects terms and the four interaction terms jointly accounted for 11.36% of the variance. The skewness of the distribution of the residuals fell in the range of normal distribution (skewness − 0.428). Homoscedasticity assumption was not violated by visual inspection. Residuals outside ± 2.5 SD occupied 1.89% of the trials (135 out of 7245) and 19 out of 20 participants in this larger data set contributed 1–23 trials (median = 3.5). When these 135 trials with outlying residuals were removed, all the significant terms remained significant and all the non-significant terms remained non-significant. Therefore, none of the results were driven by these trials. Moreover, the 10,000-times bootstrap test showed that all the significant factors and interactions were stable (Tables 8, 9 and 10; Fig. 5).

**Table 10:** Analysis 3 (n = 20, L1 and L2): back-transformed fitted reaction times (ms)

Fixed-effects levels	Fitted mean	95% CI lower bound	95% CI upper bound
First phoneme = same	935	895	979
First phoneme = different	992	947	1042
L1, log10WF = 0.06 (1.4/Mil.)	1064	1002	1134
L1, log10WF = 4 (10,000/Mil.)	904	859	953
L2, log10WF = 0.06 (1.4/Mil.)	1074	1011	1145
L2, log10WF = 4 (10,000/Mil.)	875	833	920
Run 1, log10WF = 0.06 (1.4/Mil.)	1143	1072	1225
Run 1, log10WF = 4 (10,000/Mil.)	919	873	970
Run 2, log10WF = 0.06 (1.4/Mil.)	998	943	1060
Run 2, log10WF = 4 (10,000/Mil.)	858	818	902
Run 1, trial number 2th	989	943	1039
Run 1, trial number 130th	1040	989	1096
Run 2, trial number 2nd	881	844	921
Run 2, trial number 130th	966	921	1015
Age 19, log10WF = 0.06 (1.4/Mil.)	1051	949	1179
Age 19, log10WF = 4 (10,000/Mil.)	921	842	1016
Age 32, log10WF = 0.06 (1.4/Mil.)	1088	976	1229
Age 32, log10WF = 4 (10,000/Mil.)	857	787	941

This table displays the reaction time (RT) means with upper and lower 95% confidence interval (CI) bounds as related to their respective analysis groups within analysis 3

#### Random effects

Regarding the random effects of the combined data set, the by-participant random intercept was significant (*χ*^*2*^(1) = 1630.89, *p* ~ 0, SD = 1.058e−04, 95% CI 7.147e−05, 1.398e−04). Likewise, the by-picture random intercept was significant (*χ*^*2*^(1) = 796.17, *p* = 3.664e−175, SD = 6.615-05, 95% CI 5.687e−05, 7.543e−05). Regarding the representativeness of each gender group, the by-participant random intercept of the female sample was significant (*χ*^*2*^(1) = 971.7911, *p* = 2.431e−213. Likewise, the by-participant random intercept of the male sample was significant (*χ*^*2*^(1) = 604.61, *p* = 1.662e−133. These results suggest that each gender group consisted of sufficiently diverse participants, and thus, for the final model reported below, significant effects of the gender factor and their interactions as well as other significant effects are generalizable beyond the participants in the present study.

#### Fixed effects

##### First phoneme difference

First phoneme difference was significant. It did not interact with gender or age. Reaction times were shorter for names with the same first phoneme (*M*_btf_ = 935 ms, 95% CI_btf_ 895 ms, 979 ms) than for those with the different first phonemes (*M*_btf_ = 992 ms, 95% CI_btf_ 947 ms, 1042 ms), (*b* = − 6.144e−04, *t*(7053) = − 11.291, *p* = 2.593e−29; Tables 8, 9 and 10, Fig. 5a). The direction of the difference was the same as observed in analysis 1. Thus, the effect of first phoneme difference was replicated.

##### Word frequency × language status

The word frequency × language status interaction was significant. Reaction times were shorter for high frequency names than for low frequency names. However, the advantage of higher frequency names over lower frequency names was greater in L2 (typically currently dominant-use) (L2, high frequency: *M*_btf_ = 875 ms, 95% CI_btf_ 833 ms, 920 ms; L2, low frequency: *M*_btf_ = 1074 ms, 95% CI_btf_ 1011 ms, 1145 ms) than in L1 (typically currently non-dominant use) (L1, high frequency: *M*_btf_ = 904 ms, 95% CI_btf_ 859 ms, 953 ms; L1, low frequency: *M*_btf_ = 1064 ms, 95% CI_btf_ 1002 ms, 1134 ms), (*b*_interaction_ = − 1.148e−05, *t*(6984) = 2.226, *p* = 0.026; Tables 9, 10, Fig. 5b). The pattern of the directions of the reaction time difference was the same as observed in analysis 1. Thus, the effect of the word frequency × language status interaction was replicated.

##### Word frequency × run number

The word frequency × run number interaction was significant. Reaction times were shorter for higher frequency names than for lower frequency names. However, the advantage of higher frequency names over lower frequency names was greater in run 1 (run 1, high frequency: *M*_btf_ = 919 ms, 95% CI_btf_ 873 ms, 970 ms; run 1, low frequency: *M*_btf_ = 1143 ms, 95% CI_btf_ 1072 ms, 1225 ms) than in run 2 (run 2, high frequency: *M*_btf_ = 858 ms, 95% CI_btf_ 818 ms, 902 ms; run 2, low frequency: *M*_btf_ = 998 ms, 95% CI_btf_ 943 ms, 1060 ms), (*b*_interaction_ = − 1.273e−05, *t*(7002) = − 2.456, *p* = 0.014; Tables 9, 10, Fig. 5c). The pattern of the directions of the reaction time difference was the same as observed in analysis 1. Thus, the effect of the word frequency × run number interaction was replicated.

##### Trial number × run number

The trial number × run number interaction was significant. Reaction times were longer for later trials than for earlier trials. However, the advantage of earlier trials over later trials was greater in run 2 (run 2, early trial: *M*_btf_ = 881 ms, 95% CI_btf_ 844 ms, 921 ms; run 2, later trial: *M*_btf_ = 966 ms, 95% CI_btf_ 921 ms, 1015 ms) than in run 1 (run 1, early trial: *M*_btf_ = 989 ms, 95% CI_btf_ 943 ms, 1039 ms; run 1, later trial: *M*_btf_ = 1040 ms, 95% CI_btf_ 989 ms, 1096 ms), (*b*_interaction_ = − 3.935e−07, *t*(7023) = − 3.172, *p* = 0.002; Tables 9, 10, Fig. 5d). The pattern of the directions of the reaction time difference was the same as observed in analysis 1. Thus, the trial number × run number interaction was replicated.

##### Word frequency × age

The trial number × age interaction was significant. It was a degree interaction. Reaction times were shorter for higher frequency names than for lower frequency names. This advantage of high frequency names over lower frequency names was greater for older participants (age 32, high frequency: *M*_btf_ = 857 ms, 95% CI_btf_ 787 ms, 941 ms; age 32, low frequency: *M*_btf_ = 1088 ms, 95% CI_btf_ 787 ms, 941 ms) than for younger participants (age 19, high frequency: *M*_btf_ = 921 ms, 95% CI_btf_ 842 ms, 1016 ms; age 19, low frequency: *M*_btf_ = 1051 ms, 95% CI_btf_ 949 ms, 1179 ms), (*b*_interaction_ = 2.193e−06, *t*(7020) = − 2.893, *p* = 0.004; Tables 9, 10, Fig. 5e). Thus, the word frequency effect was replicated. However, the pattern of the directions of the reaction time difference was not consistent with the prediction derived by the Weaker Links hypothesis. Thus, the Weaker Links hypothesis was not supported.

### Analysis 4 (Analysis 2 extended with n = 18)

#### Analysis 4 Overview

The data set of analysis 4 consisted of 3267 German trials from the data set of analysis 3. The data consisted of 1430 trials from eight female participants and 1837 trials from 10 male participants, and thus, it was gender-balanced.

**Fig. 6:**
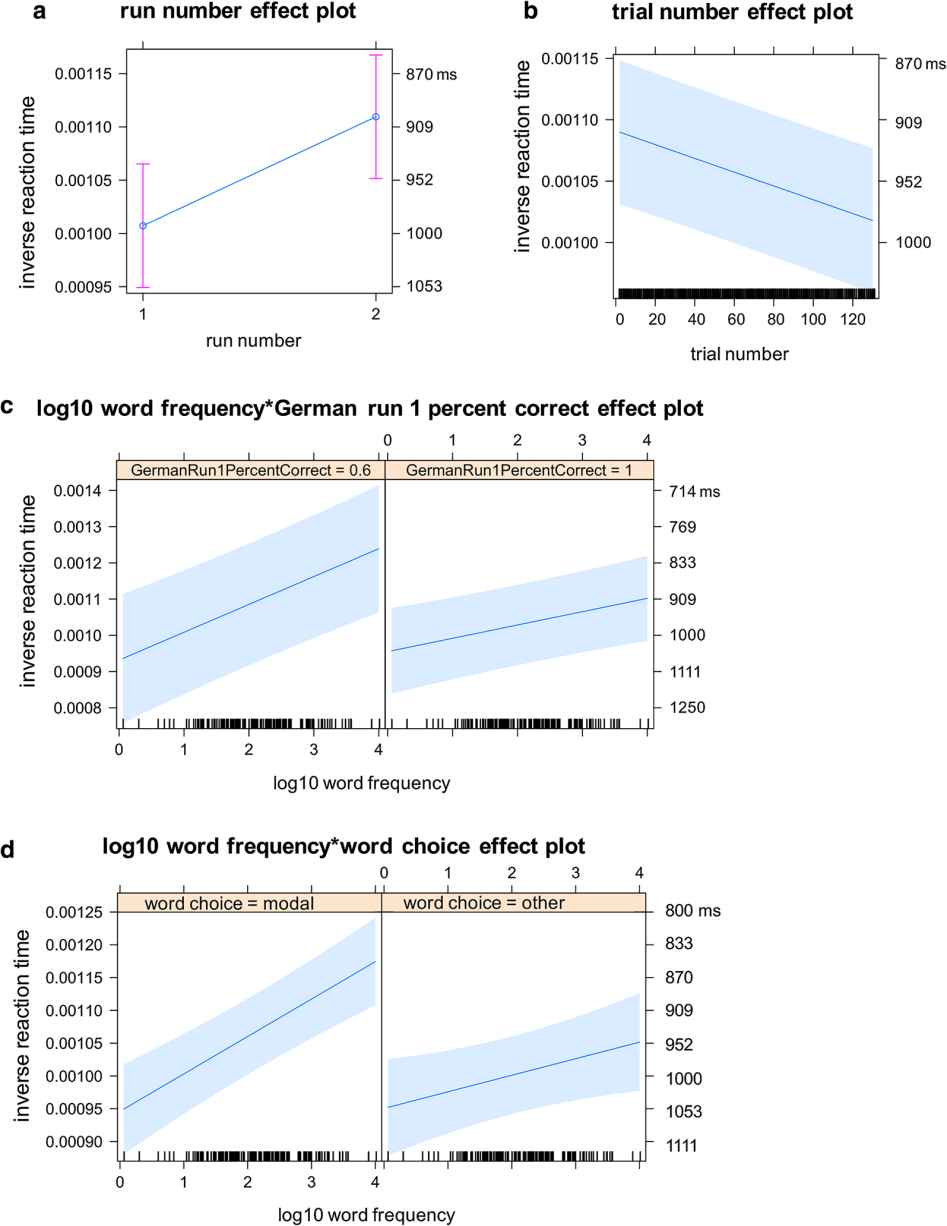
Interactions determined in analysis 4. This figure details findings made in analysis 4. This entails the replication of the effects of run number (**a**), trial number (**b**) and word frequency × German run 1 (**c**) on reaction time (RT). While the benefit of modal names over non-modal names was replicated (**d**), the difference in RT was not greater for non-modal names than for modal names

**Fig. 7:**
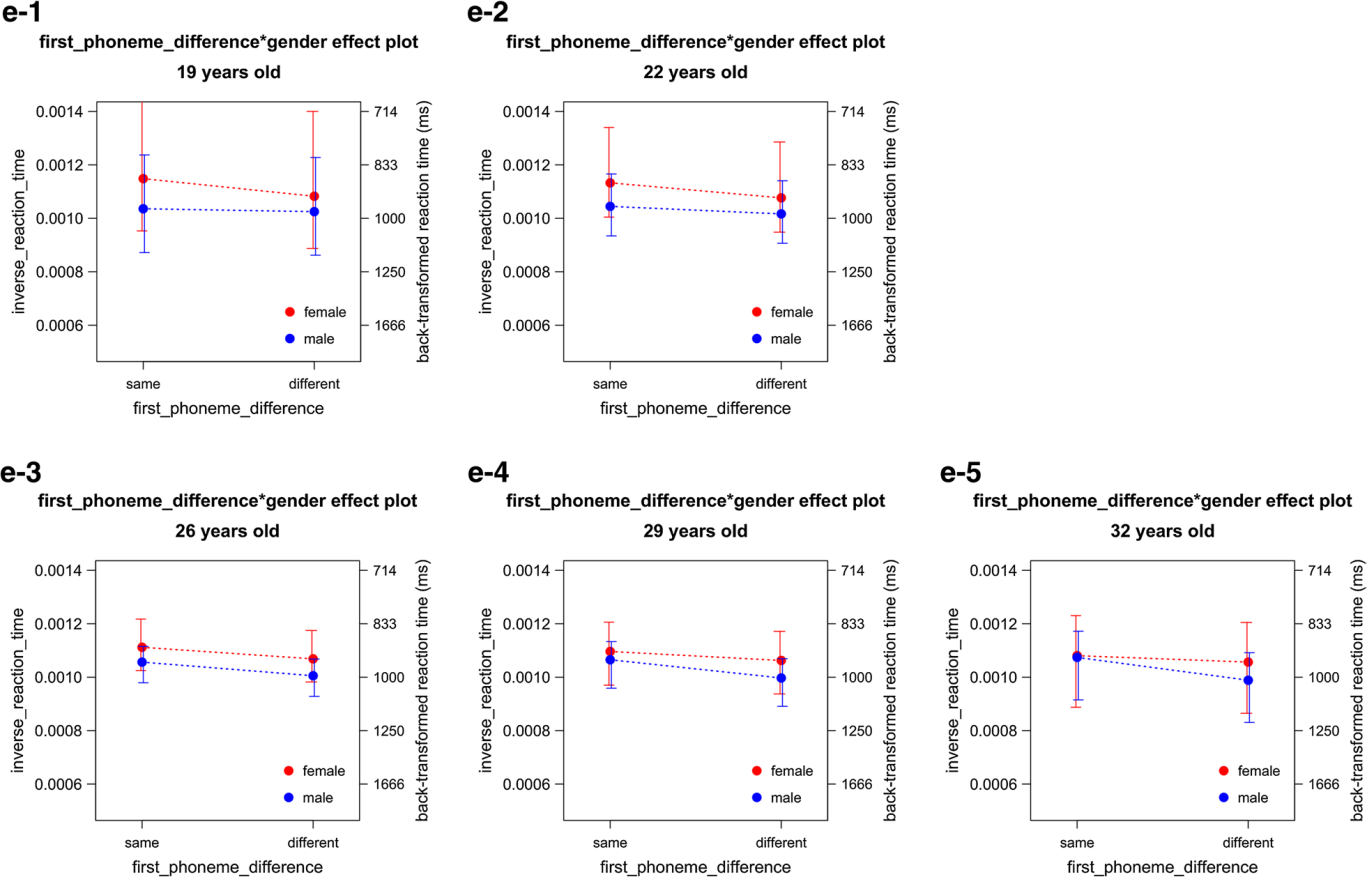
Age based modulation of gender × first phoneme interaction. This figure visualizes the effect of gender × first_phoneme_difference on reaction time (RT) split by age groups. While the facilitatory effect of shared first phoneme was for younger age groups only present in females (**e**1, **e**2), the gender difference disappeared for older age groups (**e**3–**e**5)

The final model consisted of the fixed intercept, the by-participant random intercept, the by-picture random intercept, eight fixed-effects factors, five 2-way interactions, and one 3-way interaction (Tables 11, 12 and 13, Figs. 6 and 7). Among the terms that were significant in sample 1, run number, trial number, and word frequency × German run 1 percent correct remained significant, without changing the direction of reaction time difference. Thus, each of their effects were replicated (Tables 11, 12 and 13; Fig. 6a–c). In contrast, word choice interacted with word frequency. The advantage of modal names over non-modal names was replicated. However, the reaction time difference was not greater for non-modal names than for modal names. Thus, the Cascade hypothesis was not supported (Tables 11, 12 and 13; Fig. 6d). First phoneme difference × gender interacted with age (Tables 11, 12 and 13; Fig. 7e1–e5). The superior inhibitory control of male participants decreased with the increase of age (Tables 12, 13, Fig. 7e1–e5). The pattern of first phoneme difference × gender in analysis 1 was replicated among younger participants (Tables 12, 13; Fig. 7e1, e2) but it was not replicated among the older participants (Tables 12, 13; Fig. 7e3–e5).

**Table 11:** Analysis 4 (n = 18, German only): final model by backward model comparison

Terms	Eliminated	npar	logLik	AIC	LRT	Df	p value
Fixed intercept		14	51,279.51	− 102,531.00			
(1 | partID)	0	13	50,411.47	− 100,796.90	1736.074	1	~ 0.000
(1 | picID)	0	13	50,987.03	− 101,948.10	584.9555	1	3.13E−129

row.names	Eliminated	Sum Sq	Mean Sq	NumDF	DenDF	F value	p value
firstPhonemeDiff	0	3.992E−06	3.992E−06	1	7053.577	127.485	2.593E−29
log10WF:langStatus	0	1.552E−07	1.552E−07	1	6984.862	4.955	0.026
log10WF:runNum	0	1.889E−07	1.889E−07	1	7002.267	6.031	0.014
runNum:trialNum	0	3.151E−07	3.151E−07	1	7023.822	10.060	0.002
log10WF:age	0	2.622E−07	2.622E−07	1	7020.770	8.372	0.004

This table provides a comparison of different statistical models used for analysis 4

**Table 12:** Analysis 4 (n = 18, German only): Final model table of coefficients

Terms	Estimate	Std. Error	95% CI lower	95% CI upper	t value	df	p-values
(Intercept)	1.073E−03	4.863E−04	1.053E−04	2.034E−03	2.206	14.038	0.045*
runNum2	1.023E−04	6.154E−06	9.001E−05	1.145E−04	16.615	3120.101	1.802E−59***
trialNum	− 5.613E−07	8.811E−08	− 7.388E−07	− 3.840E−07	− 6.370	3172.057	2.164E−10***
log10WF	1.435E−04	3.645E−05	7.216E−05	2.158E−04	3.937	3163.881	8.439E−05***
GermanRun1PercentCorrect	5.852E−05	3.341E−04	− 6.001E−04	7.194E−04	0.175	15.083	0.863
wordChoiceother	4.575E−06	2.117E−05	− 3.721E−05	4.597E−05	0.216	3237.562	0.829
gendermale	− 2.680E−04	4.390E−04	− 1.128E−03	5.798E−04	− 0.610	13.498	0.552
firstPhonemeDiffdifferent	− 1.269E−04	8.688E−05	− 2.930E−04	3.774E−05	− 1.461	3186.468	0.144
Age	− 5.223E−06	1.367E−05	− 3.168E−05	2.139E−05	− 0.382	13.492	0.708
log10WF:GermanRun1PercentCorrect	− 1.003E−04	4.061E−05	− 1.810E−04	− 1.937E−05	− 2.470	3158.615	0.014*
log10WF:wordChoiceother	− 3.185E−05	9.721E−06	− 5.056E−05	− 1.273E−05	− 3.276	3239.601	0.001*
gendermale:firstPhonemeDiffdifferent	2.253E−04	1.067E−04	1.798E−05	4.358E−04	2.111	3167.632	0.035*
gendermale:age	8.174E−06	1.694E−05	− 2.461E−05	4.080E−05	0.483	13.521	0.637
firstPhonemeDiffdifferent:age	3.216E−06	3.309E−06	− 3.113E−06	9.512E−06	0.972	3185.794	0.331
gendermale:firstPhonemeDiffdifferent:age	− 8.966E 06	4.079E−06	− 1.703E−05	− 1.059E−06	− 2.198	3166.559	0.028*

This table details the influence of various coefficients for the statistical model used in analysis 4

**Table 13:** Analysis 4 (n = 18, German only): back-transformed fitted reaction times (ms)

Fixed-effects levels	Fitted mean	95% CI lower bound	95% CI upper bound
Run 1	1013	964	1067
Run 2	920	879	964
Trial 2nd	918	871	970
Trial 130th	982	929	1043
GermanRun1 = 70% correct, log10WF = 0.06 (1.4/Mil.)	1062	942	1217
GermanRun1 = 70% correct, log10WF = 4 (10,000/Mil.)	830	756	920
GermanRun1 = 90% correct, log10WF = 0.06 (1.4/Mil.)	1050	974	1139
GermanRun1 = 90% correct, log10WF = 4 (10,000/Mil.)	880	827	941
Modal, log10WF = 0.06 (1.4/Mil.)	1053	983	1135
Modal, log10WF = 4 (10,000/Mil.)	851	806	903
Non-modal, log10WF = 0.06 (1.4/Mil.)	1050	975	1138
Non-modal, log10WF = 4 (10,000/Mil.)	951	888	1023
Age = 19, female, firstPhoneme = same	871	683	1049
Age = 19, female, firstPhoneme = diff	924	714	1127
Age = 19, male, firstPhoneme = same	965	808	1147
Age = 19, male, firstPhoneme = diff	976	814	1160
Age = 32, female, firstPhoneme = same	925	813	1127
Age = 32, female, firstPhoneme = diff	946	830	1156
Age = 32, male, firstPhoneme = same	931	853	1093
Age = 32, male, firstPhoneme = diff	1012	916	1204

This table displays the reaction time (RT) means with upper and lower 95% confidence interval (CI) bounds as related to their respective analysis groups within analysis 4

The extended model accounted for 47.90% of the variance. The by-participant random intercept and the by-picture random intercept jointly accounted for 33.76% of the variance. The fixed-effects terms jointly accounted for 14.14% of the variance. The skewness of the distribution of the residuals fell in the range of normal distribution (skewness − 0.335). Homoscedasticity assumption was not violated by visual inspection. Residuals outside ± 2.5 SD occupied 1.87% of the trials (61 out of 3267) and 17 out of 18 participants in the data set contributed 1–10 trials (median = 2.5). When these 61 trials with outlying residuals were removed, all the significant terms remained significant and all the non-significant terms remained non-significant. Therefore, none of the results were driven by these trials. Moreover, the 10,000-times bootstrap test showed that all the significant factors and interactions were stable (Table 12).

#### Random effects

The by-participant random intercept was significant (*χ*^*2*^(1) = 791.39, *p* = 4.023e−174, SD = 1.200e−04, 95% CI 7.395e−05, 1.662e−04). Likewise, the by-picture random intercept was significant (*χ*^*2*^(1) = 396.02, *p* = 4.04576e−88, SD = 7.021e−04, 95% CI 5.916e−05, 8.132e−05). Regarding the representativeness of each gender group, the by-participant random intercept of the female sample was significant (*χ*^*2*^(1) = 456.26, *p* = 3.128e−101). Likewise, the by-participant random intercept of the male sample was significant (*χ*^*2*^(1) = 291.9298, *p* = 1.888e−65). These results suggest that each gender group consisted of sufficiently diverse participants, and thus, for the final model reported below, significant effects of the gender factor and their interactions as well as other significant effects are generalizable beyond the participants in the present study.

#### Analysis 4 Fixed effects

##### Run number

The effect of run number was significant. Reaction times were shorter in run 2 (*M*_btf_ = 920 ms, 95% CI_btf_ 879 ms, 964 ms) than in run 1 (*M*_btf_ = 1013 ms, 95% CI_btf_ 964 ms, 1067 ms) (*b* = 1.023e−04, *t*(3120) = 16.615, *p* = 1.802e−59; Tables 12, 13; Fig. 6a). The direction of the reaction time difference was the same as observed in analysis 2. Thus, the effect of run number was replicated.

##### Trial number

The effect of trial number was significant. Reaction times were longer for later trials (trial 130th: *M*_btf_ = 982 ms, 95% CI_btf_ 929 ms, 1043 ms) than for earlier trials (trial number 2nd: *M*_btf_ = 918 ms, 95% CI_btf_ 871 ms, 970 ms) (*b* = − 5.613e−07, *t*(3172) = − 6.370, *p* = 2.614e−10; Tables 12, 13, Fig. 6b). The direction of the reaction time difference was the same as observed in analysis 2. Thus, the effect of run number was replicated.

##### Word frequency × German run 1 percent correct

The effect of word frequency × German run 1 percent correct was significant. Reaction times were longer for lower frequency words than for higher frequency words. This difference was greater for participants with lower German run 1 percent correct (70% correct, high frequency: *M*_btf_ = 830 ms, 95% CI_btf_ 756 ms, 920 ms; 70% correct, low frequency: *M*_btf_ = 1062 ms, 95% CI_btf_ 942 ms, 1217 ms) than for those with higher German run 1 percent correct (90% correct, high frequency: *M*_btf_ = 880 ms, 95% CI_btf_ 827 ms, 941 ms; 90% correct, low frequency: *M*_btf_ = 1050 ms, 95% CI_btf_ 974 ms, 1139 ms), (*b*_interaction_ = − 1.003e−04, *t*(3158) = − 2.470, *p* = 0.014; Tables 12, 13, Fig. 6c). The direction of the reaction time difference was the same as observed in analysis 2. Thus, the effect of word frequency × German run 1 percent correct was replicated.

##### Word choice and word choice × word frequency

Word choice × word frequency was significant. Reaction times were shorter for modal names than for non-modal names. The advantage of higher frequency words over lower frequency words was greater for modal names (modal, high frequency: *M*_btf_ = 851 ms, 95% CI_btf_ 806 ms, 903 ms; 70% correct, modal, low frequency: *M*_btf_ = 1053 ms, 95% CI_btf_ 983 ms, 1135 ms) than for the non-modal names (non-modal, high frequency: *M*_btf_ = 951 ms, 95% CI_btf_ 888 ms, 1023 ms; non-modal, low frequency: *M*_btf_ = 1050 ms, 95% CI_btf_ 975 ms, 1138 ms), (*b*_interaction_ = − 3.185e−05, *t*(3239) = − 3.276, *p* = 0.001; Tables 12, 13, Fig. 6d). Thus, the effect of word choice was replicated. The word choice × word frequency interaction became significant in this larger sample. However, the pattern of the directions of the reaction time difference was not consistent with the prediction by the Cascade hypothesis. Thus, the Cascade hypothesis was not supported.

##### First phoneme difference × gender × age

The effect of first phoneme difference × gender was qualified by age. Among younger participants (e.g., below 26 years old), the advantage of the same first phoneme over the different first phonemes was smaller for males (male, age 19, same first phoneme: *M*_btf_ = 965 ms, 95% CI_btf_ 808 ms, 1147 ms; male age 19, different first phonemes: *M*_btf_ = 976 ms, 95% CI_btf_ 814 ms, 1160 ms) than for females (female, age 19, same first phoneme: *M*_btf_ = 871 ms, 95% CI_btf_ 683 ms, 1049 ms; female, age 19, different first phonemes: *M*_btf_ = 924 ms, 95% CI_btf_ 714 ms, 11,217 ms, *b*_2wayInteraction_ = 2.253e−4, *t*(3167) = 2.111, *p* = 0.035; Tables 12, 13, Fig. 7e1, e2). However, among older participants (e.g., over 26 years old), the advantage of the same first phoneme over the different first phonemes increased in males (male: age 32, same first phoneme: *M*_btf_ = 931 ms, 95% CI_btf_ 853 ms, 1093 ms; male age 32, different first phonemes: *M*_btf_ = 1012 ms, 95% CI_btf_ 916 ms, 1204 ms; female: age 32, same first phoneme: *M*_btf_ = 925 ms, 95% CI_btf_ 813 ms, 1127 ms; female age 32, different first phonemes: *M*_btf_ = 946 ms, 95% CI_btf_ 830 ms, 1156 ms; *b*_3wayInteraction_ = − 8.966e−06, *t*(3166) = − 2.198, *p* = 0.028; Tables 12, 13; Fig. 7e3–e5). These results were consistent with the prediction by the decrease of the inhibitory cognitive control with the increase of age.

### Sample difference and theoretically-relevant participant-related variables

Part of the sample difference was the increase of the age range. Here we briefly report two of the age-related results that were significant in a separate comprehensive study of sample difference decomposition.

#### First phoneme difference × age of L2 acquisition

**Fig. 8:**
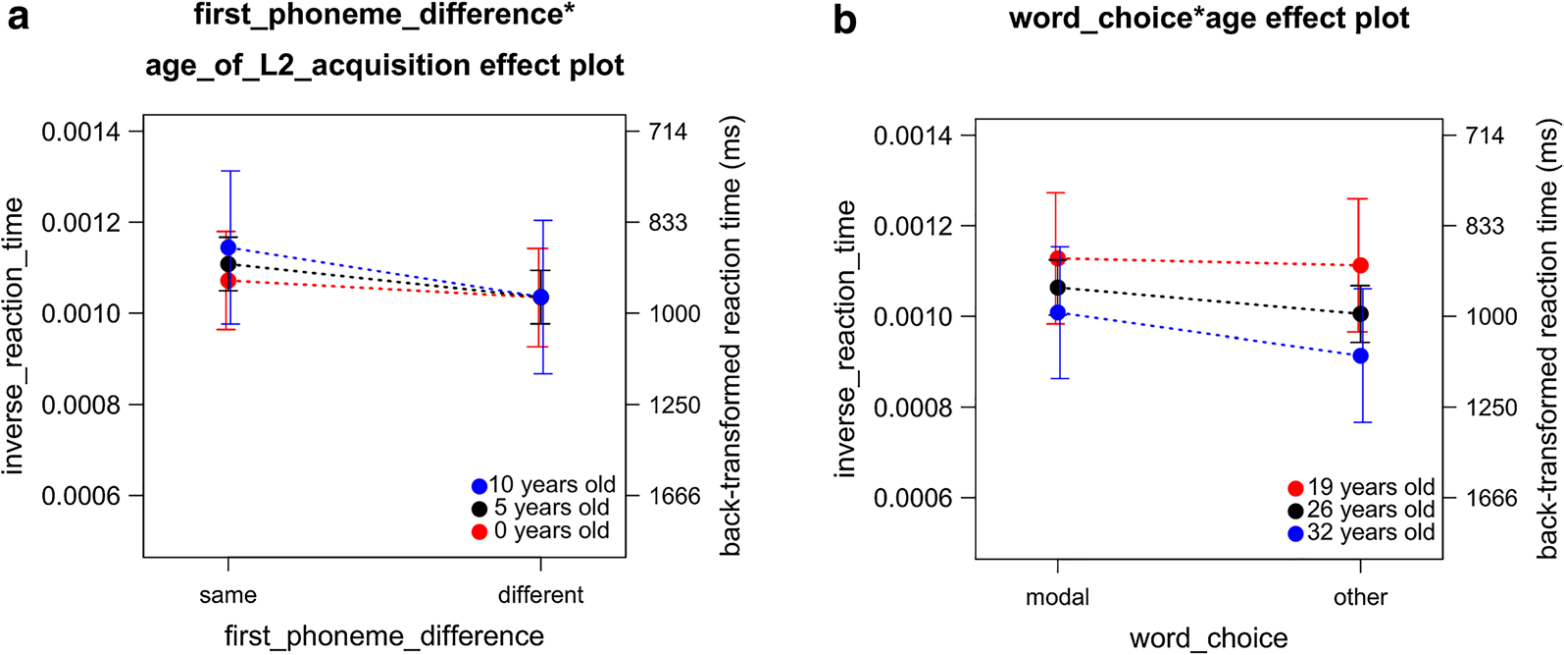
Additional interactions involving age and age of L2 acquisition. This figure shows additional findings made in analysis 3 and 4. Status of first phoneme interacts with age of L2 acquisition on reaction time (RT), whereby late-acquirers profit more from the beneficial effect of a cross-lingually shared first phoneme (**a**). Further, participant age interacts with word choice on RT, with younger participants being less held back by non-modal responses than older participants (**b**)

In a complex model to systematically decompose the sample difference present in analysis 3, first phoneme difference × age of L2 acquisition was one of the significant interactions that involved participant-related variables. The advantage of the same initial phoneme across both languages was smaller as the age of L2 acquisition was earlier (Fig. 8a). This result was consistent with the prediction derived by the different phonological encoding hypothesis.

#### Word choice × age

In a complex model to systematically decompose the sample difference present in analysis 4, word choice × age was one of the significant interactions that involved participant-related variables. The advantage of the modal names over non-modal names was smaller as the participants were younger (Fig. 8b). This result was consistent with the prediction by the decline of cognitive control with the increase of age.

## Discussion

The present study investigated in what context longer RTs for object naming are likely to occur along the various stages of single-word production in healthy proficient bilingual adults. We tested preselected factors well-established in bilingual cognition and general psycholinguistic word production theories. We also tested interactions between these factors. This could help to gain a better in toto understanding of the inter-language competition processes.

We have found that longer RTs of our proficient bilingual adults were associated with factors taken to reflect the difficulty in the semantic/lemma selection stage and the phonological code retrieval stage of single-word production interacting with cognitive states changing over trials and runs. These factors include (1) the fatigue effect building over the 131 trials for about 5 min 30 s and over 2 runs, (2) the difficulty in the semantic/lemma selection reflected in non-modal WC and the German run 1 naming accuracy, (3) the difficulty in phonological code retrieval associated with low-frequency words and words with the non-overlapping initial phoneme in the two languages, and (4) the reduced advantage of the run 2 practice effect due to the increasing fatigue effect in later trials and the minimal advantage of practice effect on high-frequency words in the second run. These findings would imply the same phenomenon to occur in settings not confined to the frame of study. Prolonged word production could, for example, play a role in the increased frequency of tip-of-tongue states for bilinguals, or possible involuntary switches between L1 and L2 partly due to exhausted executive functions [43]. This hypothesis should however be considered tentatively, because it is unclear whether exhaustion similar to the one in a test setting tends to occur outside of long and strenuous study tasks.

The most intriguing interaction was observed where phonological factors interacted with other aspects of cognitive control. The gender difference in the inhibitory control of task-irrelevant information interacted with the bilingual advantage of enhanced phonological activation from L1 to L2 shared initial phonemes, which adversely affected the male speakers. Here, their presumed superior inhibitory control suppressed the facilitative phonological activation associated with the task-irrelevant language. The female speakers on the other hand benefitted from the doubled phonological activation regarding their presumed inferior inhibitory control of the task-irrelevant information.

Another important interaction concerned the speed-accuracy tradeoff. Speakers with higher accuracy in German object naming were associated with longer RTs. Also, an interaction with WF was observed. The WF effect was smaller for slower but highly accurate participants than for quick but less accurate participants. The accuracy difference likely arises at the stage of phonological code retrieval.

### Theoretical implications

#### Support of language-independent phonological activation

In the present study, a facilitatory effect on RTs was demonstrated when both the L1 and the L2 target word shared the same initial phoneme. The presence of this effect confirms our initial hypothesis. Herein, we suspected a possible increased activation of the initial part of the target word building up by both languages providing a converging access on the level of phonological representations. As a result, a faster phonological-code retrieval process occurs compared to cases not sharing the initial phoneme. In this line, our findings support the hypothesis established by Colomé and Miozzo, which argues that during bilingual speech production, phonological representations of a given concept are activated in both languages [9, 10]. Additionally, an influence of task language status was not shown. Therefore, the lack of the language-status effect in this dataset cannot be taken as evidence for language-specific activation or the inhibitory control model [4, 11]. We suspect the lack of the language-status effect to be due to the high proficiency that our participants possess.

Additionally, we observed a significant interaction of first phoneme status with age of L2 acquisition. This falls in line with the discussion on language-independent phonological activation above, but more importantly supports the notion that age of L2 acquisition plays a role in organizing phonological representations as postulated before [24]. We can, however, make no claims regarding whether there are additional loci influenced by age of acquisition.

#### Gender difference in inhibitory control

Our working hypothesis with regards to a gender difference in inhibitory control in bilingual object naming was built on previous findings implying such a difference for certain processes relying on self-monitoring. The measure previously used was the Simon task, which requires suppressing task-irrelevant location information to correctly process task-relevant direction information and at which females were shown to perform worse [19]. While a very recent study provides compelling evidence for the case that bilingual language control is in fact isolated from other inhibitory control, such as tested in the Simon task, the possibility of an unrelated yet analogous influence of gender on language control was not addressed [44]. Thus we extended the gender difference in suppressing task-irrelevant information from the spatial domain to the language domain. Here we would, therefore, expect a gender-dependent difference in profit from other facilitating effects, such as the shared initial phoneme facilitation.

For our primary sample, the facilitatory effect of a cross-linguistically shared initial phoneme occurred in females, but not in males to the same extent. A confirmatory analysis with our secondary sample however revealed a slightly different finding, namely an interaction between first phoneme status, gender, and age. While for ages below 26 years, same initial phonemes across languages did shorten RT in women and not in men, the same was not true for ages above 26 years. We interpret this difference to signify a stronger basal level of self-monitoring about task relevance in language that is prominent in bilingual males compared to bilingual females, but is notably influenced by the worsening of cognitive control during the ageing process [21, 22]. One possible mechanism could be a stronger a priori inhibition of the non-target language, which would render any facilitation on RTs by means of a cumulative activation of phonological representation null. However, a priori inhibition of the task-irrelevant language already from the semantic process on is not consistent with our data that showed the simultaneous bilingual phonological activation.

#### Support for the weaker-links hypothesis

In our findings, the L2 responses were generally given faster than the L1 responses. This finding stands in contrast with frequent reports of the L2 being slower in word production than the L1 [5, 6]. A similar situation was reported by Christoffels and colleagues, where behavioral data showed a faster RT for the L2 than for the L1 [7]. In this study, however, the effect only occurred in language-mixed settings, whereas it disappeared in same-language block design such as the one used by us.

A possible explanation might be found in the weaker-links hypothesis, which stresses the importance of differences in WF as a highly relevant factor leading to different RTs [5, 8]. Since 75% of our participants reported German, presumably the dominant language at the time of the experiment, as their L2, the higher WF gained through the German language dominance might lead to a situation in which this paradoxical RT effect occurs. It did no escape our view however, that the WF effect showed to be stronger for the L2 as well. This in turn conflicts, on first view, the weaker-links hypothesis, which predicts that language dominance should be related to a smaller WF effect [25]. This interaction effect could be explained in two different ways.

First, it should be reminded of how the smaller WF effects is achieved along the time course of language development: WF first benefits high-frequency words in reducing RTs before low-frequency words catch up [25]. Therefore, following this line and counterfactually going back the timeline, if L1 had been the language of dominant use and L2 had been the language of non-dominant use until a point in time, RTs would have been, on average, shorter for L1 than for L2 and the WF effect would have been smaller for L1 than for L2 at that time point. Then, as L1 became the language of non-dominant use as with the bilinguals in the present study, RT increased on average for L1, keeping the previously achieved smaller WF effect for L1 but increasing the L1 RTs until RTs for L1 low-frequency word match RTs for L2 low-frequency words.

An alternative possible explanation for the conundrum of the interaction effect could come in the consideration of not only ceiling effects playing a role in activation, but also floor effects, affecting high-frequency words of non-dominant L1 adversely. There is the possibility of L1, being the predominantly non-dominant language in our dataset, summarily having reached an activation floor level through continued non-use. If in such a scenario even words with a relatively high frequency are rarely used simply due to them belonging to the L1, this attenuated activation would mean that even these high-frequency words rest on a, compared to the much more dominant L2, minor level of activation. The L2, which is summarily more activated due to its dominance, could in this context profit far more from the WF effect: only low-frequency words would rest at an activation floor, while the more often used words would experience the usual acceleration in RTs via the WF effect. This difference could explain a stronger WF effect for a dominant language; it is however a highly speculative hypothesis deserving of further critical thought.

#### Distinguishing word choice, proficiency and age of L2 acquisition

WC, proficiency in terms of naming accuracy and age of L2 acquisition are variables shown to affect semantic/lemma selection in the aforementioned studies. We intended to distinguish these variables. The choice of modal vs. non-modal responses reflects semantic decision processes at the start of word production. As expected, analysis demonstrated a significant effect of WC on RTs. Responses containing non-modal words arguably involve a more difficult semantic decision for the participant than trials in which the modal word is the obvious choice. This process of decision-making seems to take up enough time to impact the resulting RTs (by 100–200 ms on depending on word frequency). While WC does therefore still seem to be a viable measure of processing difficulty at the semantic/lemma selection stage, this study identified age as a factor that has to be taken into account. As previous studies have pointed out, bilinguals do seem to possess distinct advantages in retaining age-dependent loss of cognitive ability compared to monolinguals [21]. In this within-group setting the effects of age are still detectable, and awareness of possible confounding effects via this interaction is important.

Regarding the speed-accuracy tradeoff, our initial hypothesis concerning the inverse relationship between naming accuracy and RT speed was confirmed. As a significant main effect, a higher percentage of initially correctly named objects went in conjunction with slower RTs. In contrast to the factor of WC, naming accuracy did interact with another factor, namely WF, a variable of phonological code retrieval. Naming accuracy therefore seems to be less suited as a reflection of a purely semantic/lemma selection level than WC. This interaction could however be related to cascade models, which predict a semantic-phonological interaction. For instance, the size of the unselected semantic/lemma candidates interact with WF, which indexes phonological code retrieval. The more limited the set of candidates is, the smaller the WF effect will be [3]. Therefore, naming accuracy might be connected to a higher, task-controlling level rather than to the purely semantic/lemma selection level. From there, it would be possible for naming accuracy to influence the efficacy of word production via modulation of internal monitoring, effectively creating internal constraint on semantic/lemma selection.

This additional hypothetical link is further confirmed by the direction of the significant interaction effect that the WF effect was stronger for lower accuracy naming than for higher accuracy in our dataset. Less self-monitoring means relying more on the established activation patterns given by the WF effect, while a stronger monitoring results in a stricter internal constraint with less reliance on established activation levels. This pattern falls in line with previous research, showing an inverse relationship between semantic constraint and WF effect in object naming predicted by cascade models [3]. If we hypothesize that naming accuracy is part of the higher-level constraint generating system, it remains to be seen, in future studies, specifically on what aspect the naming accuracy variable imposes a top-down constraint. Here, the soon to be made available name- and image-agreement rating scores specific to our set of objects will certainly prove to be helpful.

Age of acquisition did not turn out to be a significant factor on the level of semantic/lemma selection in our study. We conclude that for the purpose of reflecting semantic processing, WC is the most well-suited variable in the present study [5].

### Limitations

Data analysis under factorial study designs with analysis of variance without the use of mixed-effects multiple regression usually requires a very extensive set of data. Considering the huge sample sizes common for variance-based analyses, we have to acknowledge that our small sample size limits our interpretations.

We circumvented this by taking advantage of the flexibility that the mixed-effects multiple regression analysis offers but that the conventional analysis of variance does not. By using mixed-effects multiple regression, the present study detected the effects of 10 theoretically motivated categorical factors and continuous factors and their interactions on trial-by-trial RT measured for 7145 trials for analysis 1 and 3267 trials for analysis 2. In addition to the advantage of multiple regression analysis that is able to compute the effects of fixed-factors, controlling for all other factors in the model, mixed-effects multiple regression performs by-participant analysis and by-item analysis standardly required from psycholinguistic study in one analysis and partialled out the significant participant-random effect (idiosyncrasy of the study participants) and the significant item-random effect (idiosyncrasy of objects used in the study); thus, the significant effects of the fixed factors should be generalizable to people and stimuli outside the samples used in the study.

Furthermore, our data is subject to an imbalanced language distribution. 75% of our participants reported German as their L2, which may be enough to heavily influence the results, but not enough to clearly attribute any specific observations to. This imbalance would pose a problem if the statistical method was insufficient to partial out the effects of other fixed factors and random effects of participants and items. The consequences might include:

- 25% non-dominant L2 masking an even stronger WF effect for L2, which could, if present, be interpreted to disconfirm the weaker-links hypothesis.
- 15% dominant L1 feigning a bigger WF effect for the L1. If this were the case, it might also be interpreted against the weaker-links hypothesis.
- Skewing of RT towards a German language-specific average, weakening the potential for generalization of our data interpretations [45].

Outside statistics, regarding language dominance, we assume German language dominance due to the experiment taking place in a German-speaking frame, yet there was no specific data lifted regarding the amount of usage of each participant’s languages.

Similarly, because the source study for which we measured object naming RT does not have supplementary language proficiency scores measured on established batteries in languages of the participants (German, English, French, Italian, Luxembourgian, Slovakian, Chinese, Bosnian, Croatian, Spanish, and Cantonese) beyond object naming accuracy, we can make no hard statements regarding individual language proficiency, a factor that has been suspected to strongly influence bilingual word production peculiarities [7, 46].

Lastly, we have to concede that for variables such as gender, it is impossible for us to control for any unknown third factors across the grouping variable. To solve this problem, a much bigger sample size across many different personal backgrounds would be required, which we unfortunately did not have access to.

## Conclusions

Our mixed-effects multiple regression analysis of bilingual object naming RT revealed that the single word production process in healthy adult bilinguals is affected by interactions among cognitive, phonological, and semantic factors. Bilingual phonological activation interacted with gender in the inhibitory control of task-irrelevant language. Phonological code retrieval interacted with language status, language dominance, practice effect and speed-accuracy tradeoff. The practice and fatigue effects interacted as well. Age of acquisition appears to modulate phonological word representations. Our analysis revealed that WC stands out as a robust predictor, unaffected by other factors, to detect failures in semantic/lemma selection. Taken together, dense interactions between phonological factors and other factors revealed in the present study have confirmed that meaning-sound mappings are arbitrary within and across different languages and bilingual brains orchestrate cognitive, psycholinguistic, and functional components to enable speedy and accurate single word production.

## Data Availability

The datasets used and/or analysed during the current study are available from the corresponding author on reasonable request.

## Abbreviations

CI: confidence interval
NR: no response
nTMS: navigated transcranial magnetic stimulation
L1: first language
L2: second language
PE: performance error
RT: reaction time
SD: standard deviation
SE: semantic error
WC: word choice
WD: word duration
WF: word frequency
